# Quantitative Features Analysis of Water Carrying Nanoparticles of Alumina over a Uniform Surface

**DOI:** 10.3390/nano12050878

**Published:** 2022-03-06

**Authors:** Abdul Hamid Ganie, Fazlullah Fazal, Carlos Andrés Tavera Romero, Muhammad Sulaiman

**Affiliations:** 1 Basic Science Department, College of Science and Theoretical Studies, Saudi Electronic University, Abha Male 61421, Saudi Arabia; 2 Department of Mathematics, Abdul Wali Khan University, Mardan 23200, Pakistan; ffazal1997@gmail.com; 3 COMBA R&D Laboratory, Faculty of Engineering, Universidad Santiago de Cali, Cali 76001, Colombia; carlos.tavera00@usc.edu.co

**Keywords:** Levenberg-Marquardt algorithm, backpropagation neural network, mathematical modeling, Runge-Kutta order four technique, machine learning, water and alumina nanofluid, volume fraction

## Abstract

Little is known about the rising impacts of Coriolis force and volume fraction of nanoparticles in industrial, mechanical, and biological domains, with an emphasis on water conveying 47 nm nanoparticles of alumina nanoparticles. We explored the impact of the volume fraction and rotation parameter on water conveying 47 nm of alumina nanoparticles across a uniform surface in this study. The Levenberg–Marquardt backpropagated neural network (LMB-NN) architecture was used to examine the transport phenomena of 47 nm conveying nanoparticles. The partial differential equations (PDEs) are converted into a system of Ordinary Differential Equations (ODEs). To assess our soft-computing process, we used the RK4 method to acquire reference solutions. The problem is investigated using two situations, each with three sub-cases for the change of the rotation parameter K and the volume fraction ϕ. Our simulation results are compared to the reference solutions. It has been proven that our technique is superior to the current state-of-the-art. For further explanation, error histograms, regression graphs, and fitness values are graphically displayed.

## 1. Introduction

The Coriolis force causes gases and liquids in the northern hemisphere to bend to the right. The Coriolis force plays a significant role in fundamental flow equations as an inertial force, magnetohydrodynamic force, and viscous force. Each fluid that moves on the earth’s surface is subjected to gravitational force, pressure gradient force, centrifugal force, and frictional force. While the Coriolis force does not affect every flow, it does have the power to shift the direction of transportation phenomena on the earth’s surface in the atmosphere and ocean. As a result, accepting that the Coriolis force has no influence on any non-static transport phenomena on the earth’s surface is impractical.

In addition to the Coriolis effects, the earth’s rotation has a major role in global climate change. According to [1,2], the Coriolis force may be used to characterize the whirling directions of typhoons, hurricanes, strong cyclonic storms, and strong cyclonic storm surges. In [3], researchers present an analysis of layer flow with an integral boundary of an incompressible, constant momentum in three dimensions, with a focus on the importance of rotation as it applies to the blades of a wind turbine. One of the findings was that, in addition to centrifugal forces, Coriolis forces also play a role in transport phenomena. The radial flow observed in the boundary layer is caused by centrifugal acceleration, whereas the flow in the clockwise direction is caused by the Coriolis force.

The Coriolis force is employed in businesses such as photobioreactors, sewage treatment, and bioreactors for particular tissues; see [1,2]. In [3] researchers provide a three-dimensional analysis of an incompressible constant momentum applied to wind turbine blades, with an emphasis on the relevance of rotation. The Coriolis force and the centrifugal force are both responsible for radial flow in the boundary layer. On the other hand, only the Coriolis force is responsible for flow in the clockwise direction. The Coriolis effects on the dynamics in the centrifugal bioreactor were investigated by [4], and it was observed that as the rotation of the flow increases, vortices develop and that the dynamics are regulated by Coriolis force. Coriolis forces strongly impact smaller microbial cultures because they become locked in vortices. On the other hand, large cells have larger rates of sedimentation, resulting in streamline deflection and continuing Coriolis pressures. However, this results in a stably fluidized bed of cells. In the field of atmospheric science, researchers are experimenting with several forms of nanoparticles to remediate contaminated waterways [5]. The influence of various fluids with different thicknesses on the dynamics of the Coriolis force on an object, such as the sharp edge of an airplane or rotating automobiles, was recently explored by [6]. Internal shear stress may be reduced by increasing the flow’s maximum velocity, whereas buoyant forces can raise the flow’s total velocity. The shear stress will be calculated less as the rotation across the flow increases.

Term nanofluid is formed by [7] to designate fluid substances suitably combined along with particles whose diameter falls within the nanoscale range. Particles with a diameter of less than 100 nm have greater thermal, chemical, mechanical, optical, electrical, and magnetic capabilities than regular solids due to their large ratio of surface-area-to-volume. Industries confront challenges such as cooling concerns and product maintenance due to rising heat generation. However, scientists have come to appreciate the thermal and particular nature properties of various fluids generated by solid particles addition (inn micrometer or millimeter scales) to protect energy and save procedure time.

Nanofluids are employed in a wide range of applications in industries, comprising coolants for car engines, cancer therapies, delivery of nano-drugs, syphilis testing, and detergent having nanofluid [8]. The effect of size of the particles on the dynamic viscosity of water–47 nm Al${}_{2}$O${}_{3}$, water–29 nm CuO and water–36 nm Al${}_{2}$O${}_{3}$ is given by [9]. When particle volume fractions are less than 4%, then the viscosities of both nanofluids containing Al${}_{2}$O${}_{3}$ are equivalent. There are various small or no facts about heat sinks’ influence on the nanofluid movement in thermal management. Moreover, how to retain the temperature at the heat sink’s base as low as possible while the heat transfer rate increases are discussed in [10].

In comparison to nanofluid CuO–water and distilled water, results demonstrate that nanofluid Al${}_{2}$O${}_{3}$–water has a higher transfer rate of heat. Furthermore, the heat sink can reduce generated heat by 89.6% between the mini-channels. The dynamics of water transporting titania copper and nanoparticles of alumina through a cylinder that is shaped like a wave is investigated by [11]. Volume fraction at all levels, this was revealed that friction at the wall and the local skin friction are negligibly proportional during the motion of water transporting alumina nanoparticles. Nanoparticles addition to the wall can dramatically limit the rate of heat transfer; see [12]. The rising property of random movement of particles having nano-sized mixed thoroughly with base fluid is the distribution of temperature through the nanofluid dynamics; see [13]. In [14,15], the effect of increasing Coriolis force of non-Newtonian Casson fluid on dynamics in the absence and presence of Lorentz force was investigated. The impacts on dynamics of water transporting 47 nm nanoparticles of alumina, Coriolis force, volume fraction, and heat source/sink over uniform surfaces have not been studied. Following up the literature on the findings mentioned above, it is critical to find scientifically valid solutions to the following questions.

1. What effect does Coriolis force have on layer flow at the boundary of a nanofluid alumina-water subjected to heat?
2. How does the growing volume percentage of nanoparticles impact the wall friction, as well as the mass and heat transfer rates in a nanofluid of alumina–water?
3. How does the volume percentage of 47 nm alumina nanoparticles affect heat mass transfer rate and local skin friction?
4. How can numerical solutions to the model be calculated with accuracy and efficiency. Nowadays, in the modern world, we need a method that requires less effort while giving good efficiency.
5. How can we get a simple and easy method which gives accurate results?
6. How can we save our time?

The rest of the manuscript is organized such that in Section 2 a detailed problem formulation is elaborated. In Section 3, the solution methodology is presented. Results and discussions are given in Section 4. At the end of this manuscript, we present the conclusions of this study.

## 2. Formulation of the Principal Equations

The derivation is presented for transport phenomena principal equations and the inertial forces of a frame of reference that is rotating.

### 2.1. Forces for a Frame of Reference Which Is Rotating

The planned path and specious route are two visual pathways in a frame of reference with rotation. In the true/intended path, the trajectory is effortlessly warped by a fake force recognized as the Coriolis force, resulting in the specious path. Suppose the angular velocity Ω, position vector $r=(r_{1},r_{2},r_{3})$ and in the rotating frame the absolute time derivative:

(1) $$\frac{Dr}{Dt}=\frac{dr}{dt}+\Omega \times r,\;\frac{dr}{dt}=(\frac{dr_{1}}{dt},\frac{dr_{2}}{dt},\frac{dr_{3}}{dt}).$$

According to the second law of motion of Newton, force is:

(2) $$F=ma_{r}-\frac{d\Omega }{dt}m\times r-2m\Omega \times \frac{dr}{dt}-m\Omega \times \left(r\times \Omega \right).$$

Euler force are the three acting principal forces on a body travelling in such a frame which is rotating in Equations (1) and (2). $F_{e}=-m\frac{d\Omega }{dt}\times r$, Coriolis force $F_{c}=-2m\Omega \times \frac{dr}{dt}$, and Centrifugal force $F_{g}=-m\Omega \times \left(r\times \Omega \right)$. Azimuthal force (also known as Euler’s force) is required to keep the body in orbit because it acts parallel to but against the angular velocity. The centrifugal force acts radially outward from the axis of rotation of the rotating frame. The angular velocity and the Coriolis force are perpendiculars, and the Coriolis force acts outward. After combining the Navier–Stokes equation published by [4,6], the magnitude order was executed on $\frac{dr}{dt}$ for boundary layer flow. The buoyancy and Coriolis forces on the flow of fluid in a uniformly thick horizontal surface in x and z axes are referred to as the body force term.

(3) $$\begin{array}{cc} \; & f_{bx}=g\beta (T-T_{\infty })+g\beta^{*}(C-C_{\infty })-2\Omega w, \end{array}$$

(4) $$\begin{array}{cc} \; & f_{bz}=g\beta (T-T_{\infty })+g\beta^{*}(C-C_{\infty })+2\Omega u. \end{array}$$

The rotating frame’s angular velocity is Ω, volumetric thermal expansion is β, gravity is *g*, and β is the nanofluid’s thermal expansion coefficient. Given that the length of characteristics *ℜ* is measured in meters, the unit of the body forces fbx and fbz is m s${}^{-2}$.

### 2.2. Mathematical Formulation

**Figure 1:**
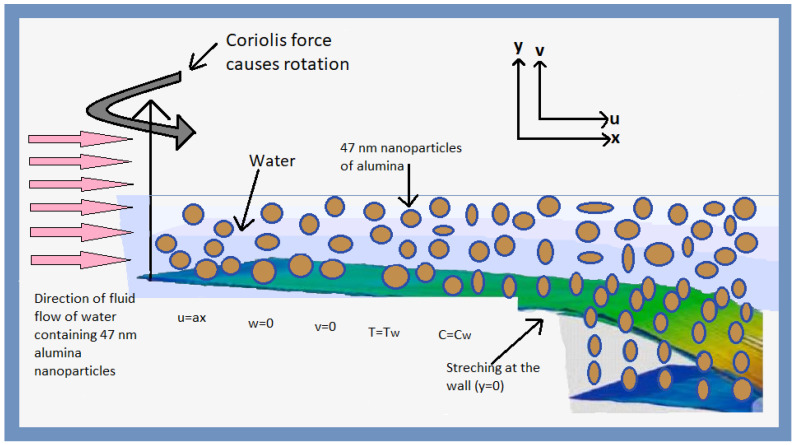
Graphical representation of the water having 47 nm nanoparticles of alumina of the transport phenomenon.

Alumina–water nanofluid dynamics on a rotating horizontal surface at a fixed time with varying thicknesses are investigated. Figure 1 shows how the surface rotates counter-clockwise. The dynamics of an alumina–water nanofluid on a rotating horizontal surface at a fixed time and with varying thicknesses are investigated [16]. Figure 1 shows how the surface rotates counter-clockwise. As a result, regulating of flow by boundary layer equation in the presence of Coriolis force are shown in Figure 1 is:

(5) $$\begin{array}{cc} \; & \frac{\partial u}{\partial x}+\frac{\partial v}{\partial y}=0, \end{array}$$

(6) $$\begin{array}{cc} \; & u\frac{\partial u}{\partial x}+v\frac{\partial u}{\partial y}=\frac{\mu_{nf}}{\rho_{nf}}\frac{\partial^{2}u}{\partial y^{2}}+\left(T-T_{\infty }\right)g\beta +(C-C_{\infty })g\beta^{*}-2\Omega w, \end{array}$$

(7) $$\begin{array}{cc} \; & u\frac{\partial w}{\partial x}+v\frac{\partial w}{\partial y}=\frac{\mu_{nf}}{\rho_{nf}}\frac{\partial^{2}w}{\partial y^{2}}+g\beta (T-T_{\infty })+g\beta^{*}(C-C_{\infty })+2\Omega u, \end{array}$$

(8) $$\begin{array}{cc} \; & u\frac{\partial T}{\partial x}+v\frac{\partial T}{\partial y}=\alpha_{nf}\frac{\partial^{2}T}{\partial y^{2}}+\tau [\frac{D_{B}}{\Delta C}\frac{\partial C}{\partial y}\frac{\partial T}{\partial y}+\frac{D_{T}}{T_{\infty }}\frac{\partial T}{\partial y}\frac{\partial T}{\partial y}]+\frac{Q_{o}[T_{w}\left(x\right)-T_{\infty }]}{{\left(\rho C_{p}\right)}_{nf}}\mathrm{Exp}[-ny\sqrt{\frac{a}{\vartheta_{bf}}}], \end{array}$$

(9) $$\begin{array}{cc} \; & u\frac{\partial C}{\partial x}+v\frac{\partial C}{\partial y}=D_{B}\frac{\partial^{2}C}{\partial y^{2}}+\frac{D_{T}\Delta C}{T_{\infty }}\frac{\partial^{2}T}{\partial y^{2}}, \end{array}$$

the following are the boundary conditions:

(10) $$\begin{array}{cc} \; & \;v=0,u=ax,\;w=0,\;C_{w}=C,\;T=T_{w},\;\mathrm{at}\;y=0, \end{array}$$

(11) $$\begin{array}{cc} \; & u\to 0,\;w\to 0,\;T_{\infty }=T,\;C_{\infty }=C\;\;\mathrm{when}\;y\to \infty . \end{array}$$

The components of velocity are u in the x-direction and v in the y-direction, the concentration of expansion coefficient is $\beta^{*}$, free stream temperature $T_{\infty }$, nano fluid temprature *T*, nanofluid kinematic viscosity is $\vartheta_{n}f$, the temperature of wall is $T_{w}$, nanofluid concentration is *C*, the concentration at free stream $C_{\infty }$, wall concentration $C_{w}$, the volume fraction of nanoparticle ϕ, coefficient of thermophoretic diffusion $D_{T}$, coefficient of Brownian diffusion $D_{B}$, angular velocity Ω, nanofluid thermal diffusivity is $\alpha_{nf}$, the thermal conductivity of nanofluid $k_{nf}$, nanofluid specific heat capacity (${\rho c\rho )}_{nf}$, and the ratio of specific heat capacity of the base fluid to the specific heat capacity of the nanoparticle is $\tau =\frac{{\left(\rho c_{p}\right)}_{np}}{{\left(\rho c_{p}\right)}_{bf}}$. Following [17,18,19,20], the density, thermal conductivity, similarity variables, and specific heat capacity of the nanofluid are specified as:

(12) $$\begin{array}{c} \alpha_{nf}=\frac{\kappa_{nf}}{{\left(\rho c_{p}\right)}_{nf}},\;\kappa_{nf}=\kappa_{bf}[\frac{\kappa_{np}+2\kappa_{bf}-2\phi (\kappa_{bf}-\kappa_{np})}{\kappa_{np}+2\kappa_{bf}+\phi (\kappa_{bf}-\kappa_{np})}],\;\rho_{nf}=\left(1-\phi \right)\rho_{bf}+\phi \rho_{np},\\ {\left(\rho c_{p}\right)}_{nf}=\left(1-\phi \right){\left(\rho c_{p}\right)}_{bf}+\phi {\left(\rho c_{p}\right)}_{np},\;\frac{\mu_{nf}}{\rho_{nf}\nu_{bf}}=\frac{0.904e^{0.148\phi }}{1-\phi +\frac{\rho_{np}}{\rho_{bf}}\phi },\;v=-{\left(a\vartheta_{bf}\right)}^{1/2}F\left(t\right),\\ u=ax\frac{dF}{dt},w=\mathrm{axH}\left(t\right),\;t=y\sqrt{\frac{a}{\vartheta_{bf}}},\;\Phi =\frac{C-C_{\infty }}{C_{w}-C_{\infty }}\;\Theta =\frac{T-T_{\infty }}{T_{w}-T_{\infty }}. \end{array}$$

Obtaining the dimensionless equations as:

(13) $$\begin{array}{c} \frac{\mu_{nf}}{\rho_{nf}\nu_{bf}}F^{\prime \prime }+FF^{\prime \prime }-F^{\prime }F^{\prime }-KH+Gr_{t}\Theta +Gr_{np}\Phi =0, \end{array}$$

(14) $$\frac{\mu_{nf}}{\rho_{nf}\nu_{bf}}H^{\prime \prime }+H^{\prime }F-HF^{\prime }+KF^{\prime }+Gr_{t}\Theta +Gr_{np}\Phi =0,$$

(15) $$\frac{\left[\frac{k_{np}+2k_{bf}-2\phi (k_{bf}-k_{np})}{k_{np}+2k_{bf}+\phi (k_{bf}-k_{np})}\right]}{\left(1-\phi \right)+\phi \frac{{\left(\rho c_{p}\right)}_{np}}{{\left(\rho c_{p}\right)}_{bf}}}\Theta^{\prime \prime }+N_{b}\Theta^{\prime }\Phi^{\prime }+N_{t}\Theta^{\prime }\Theta^{\prime }+\mathrm{PrF}\Theta^{\prime }+\mathrm{Pr}\gamma \frac{\mathrm{Exp}(-n\eta )}{\left(1-\phi +\phi \frac{{\left(\rho C_{p}\right)}_{sp}}{{\left(\rho C_{p}\right)}_{bf}}\right)}=0,$$

(16) $$\Phi^{\prime \prime }+\frac{N_{t}}{N_{b}}\Theta^{\prime \prime }+ScF\Phi^{\prime }=0,$$

where

(17) $$\begin{array}{cc} \; & \gamma =\frac{Q_{o}}{{\left(\rho C_{p}\right)}_{bf}a},\;K=\frac{2\Omega }{a},\;Gr_{t}=\frac{g\beta (T_{w}-T_{\infty })}{a^{2}x},\;Pr=\frac{\vartheta_{bf}}{\alpha_{bf}},\\ \; & N_{b}=\frac{\tau D_{B}}{\alpha_{bf}},\;N_{t}=\frac{\tau D_{T}(T_{bf}-T_{\infty })}{T_{\infty }\alpha_{bf}},\;Gr_{np}=\frac{g\beta^{*}(C_{w}-C_{\infty })}{a^{2}x},\;Sc=\frac{\vartheta_{bf}}{D_{B}}, \end{array}$$

are the thermal Grashof number, rotation parameter, Brownian parameter, Schmidt number, thermophoretic parameter, nanoparticle Grashof number, and Prandtl number. The following are the non-dimensionalized boundary conditions:

(18) $$\begin{array}{cc} \; & \;F=0,F^{\prime }=1,\;H=0,\;\Theta =1,\;\Phi =1\;\;\mathrm{at}\;t=0, \end{array}$$

(19) $$\begin{array}{cc} \; & F^{\prime }\to 0,\;H\to 0,\;\Theta \to 0,\;\Phi \to 0,\;\;\mathrm{as}\;t\to \infty . \end{array}$$

The thermophysical properties of the water and 47 nm alumina nanoparticles are essential to remember, which are given as:

(20) $$\begin{array}{c} k_{np}=40\;W\;m^{-1}K^{-1},\;k_{bf}=0.613\;W\;m^{-1}K^{-1},\;{\left(\rho cp\right)}_{np}=3,037,050\;J\;K^{-1}m^{-3},\\ \;{\left(\rho cp\right)}_{bf}=4,166,880.90\;J\;K^{-1}m^{-3},\rho_{np}=3970\;K\;g\;m^{-3},\;\mathrm{and}\;\rho_{bf}=997.10\;K\;g\;m^{-3} \end{array}$$

see Nehad et al [21]. First, we get the solutions of the problem by Mathematica using Rk4 command, and then we apply neural on that data and get the outputs of the neural using

(21) $$\begin{array}{c} Gr_{t}=1,Gr_{np}=1,N_{b}=N_{t}=0.1,n=1,Sc=0.62. \end{array}$$

Through transformation first, we obtained the Ordinary Differential Equations (ODEs) system of the considered problem and solved the system. In this paper, we solve the system of obtained Ordinary Differential Equations (ODEs) and then get the solution of the system at 1001 different points. Then, we discuss the effect of two other parameters i.e., rotation parameter K and the volume fraction ϕ, on the given problem. We also solve the system through neural networks and compare the values with the reference solution using different values of different parameters. We discuss the effects of rotation and volume fraction parameters on the system using a neural network. The heat transfer rate is proportional to the Nusselt number, and the mass transfer rate of the nanofluid of the problem is proportional to the Sherwood number.

## 3. Solution Methodology

We use artificial neural networks in this article due to the important individual qualities of this method. Artificial neural networks avoid singularity; if there is any singularity present in the equations, the neural networks avoid it and give good results [22,23]. A neural network provides a general solution to the problem, due to which we can find values of the problem outside the interval as well [24]. The significance of the neural network approach to mainly non-linear modeling capability, likely interpolations, forecasting, extrapolations, ill-conditioning, robustness to noise and insufficient data, and having ease of use, are discussed in [25]. It also has good fault tolerance ability [26]. NN can train the machine itself; that is, ANNs learn from the events and apply those properties on similar events [27]. NN has the property to perform different functions at the same time [28]. Neural networks (NN) are deep learning algorithms. Neural networks are machine learning networks, and the human brain inspires these networks. Neural networks contain one input layer, hidden neurons, and one or more than one output layer. The hidden neurons are connected and pass the signals between one another. The artificial neural network (ANN) is an algorithm used for non-linear training. It is essential due to its fast converging speed and accurate results. Performance achieved by this method is outstanding.

The Levenberg–Marquardt neural network consists of structure layer, the number of hidden neurons, network topology, and selection of input data and output data arbitrary for training, validation, and testing samples. All these things need to be determined for the solution of a problem. In this system of ODEs, we used 1001 points obtained by using the “NDSolve” command in Mathematica by using the Runge-Kutta order-4 method. We set the step size 0.004 for getting the 1001 points between 0 and 4. Then, we use those data set points in the neural toolbox by using the Matlab command window and suggest the LMB-NN method for the best result, and then we set input and output in LMB-NN. We choose the following set of data in this problem.

- Data for training is 90 percent.
- Data for testing is 5 percent.
- Data for validation is 5 percent.

**Figure 2:**
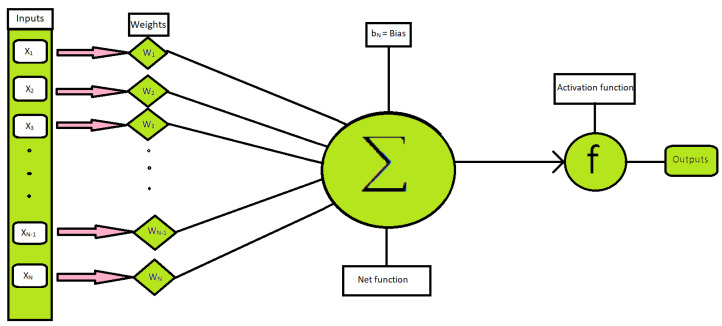
Basic design of a neural network.

Choosing as in Figure 2 the number of hidden neurons is arbitrary, and we choose in our problem the number of hidden neurons is 40, which gives us good results.

Figure 2 shows us the basic working phenomenon of a neural network that how ANN works. We see that the neural network consists of inputs and the weights assigned to these inputs, then the hidden neuron, activation function, and in the last, it gives us the outputs.

**Figure 3:**
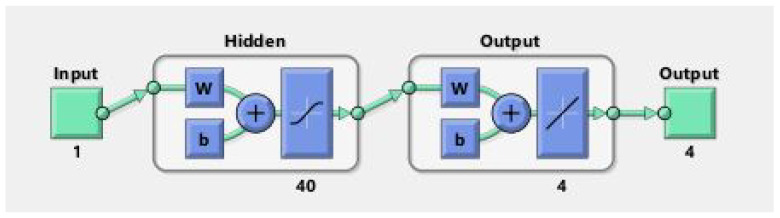
Showing the model which is generated through nftool command in matlab window.

The model we obtained for our problem of the neural networks using the nftool command in the Matlab window is shown in Figure 3. This problem contains 40 neurons. You may change the number of neurons, but the 40 neurons give us the best results in this problem. That is why we use 40 neurons in our problem. The inputs and outputs can also be clearly seen from Figure 3.

First, the partial differential equations are transformed into the system of ordinary differential equations. Then, one must solve those systems of ordinary differential equations in Mathematica using RK4 technique and obtain the system’s solution on 1001 different points by using 0.004 step size. Then, this data at 1001 points are copied to an Excel sheet, and from the MS Excel sheet, the data are transferred to Matlab for using the neural networks. In the neural networks, LMB-NN chooses for the solution of the system. In this system, there are four input values, four hidden layers, and four output values, as shown in Figure 3. The number of hidden neurons chosen is 40. Ninety percent values are selected for training, five percent for testing, and five percent is for validation. Run the command for several iterations. If the output values obtained are excellent and accurate, the process is ended; otherwise, it is necessary to retrain the network and run again until it reaches the minimum mean square error value (MSE). Depending which iteration the network gives better outputs, that will be the stopping iteration.

## 4. Results and Discussion

This study examined the effects of volume fraction, heat sink, and Coriolis force on the behavior of water conveying 47 nm alumina nanoparticles over a uniform surface using a soft computing technique. To generate nanofluids, researchers used Al${}_{2}$O${}_{3}$ nanoparticles ranging in size from 13 nm to 302 nm, with a 2 percent to 36 percent increase in thermal conductivity (see [29]). As a result of ever-increasing heat production, industries face cooling issues and product maintenance obstacles. However, scientists appreciate the precise nature and thermal properties of various fluids generated by adding solid particles (on the micrometer and millimeter scales) to reduce energy consumption and processing time. Nanofluids are employed in a variety of applications, including automotive engine coolants, cancer therapies, nano-drug delivery, syphilis diagnosis, and detergent with nanofluids (see [8]). Particles with less than 100 nanometers diameter have better mechanical, thermal, optical, magnetic, chemical, and electrical properties than ordinary solids (see [16]). Alumina nanoparticles with a diameter of 47 nanometers are suitable in the above applications because they have a substantial surface-area-to-volume ratio. Furthermore, Ali et al. (see [10]) discussed how to keep the temperature at the heat sink’s base as low as possible as the heat transfer rate increases. In comparison to CuO–water nanofluid and distilled water situations, the results demonstrate that Al${}_{2}$O${}_{3}$–water nanofluid has a higher heat transfer rate. Furthermore, the heat sink can reduce generated heat by 89.6% between the mini-channels. At all levels of volume fraction, it was revealed that local skin friction proportional to friction at the wall is negligible during the motion of water transporting alumina nanoparticles. The effect of particle size on the dynamic viscosity of Nguyen et al. [9] described the effect of particle size on the dynamic viscosity of:

(a) Water–36 nm Al${}_{2}$O${}_{3}$;
(b) Water–47 nm Al${}_{2}$O${}_{3}$; and
(c) Water–29 nm CuO nanoparticles experimentally.

When the particle volume fraction is less than 4%, the viscosities of both nanofluids with Al${}_{2}$O${}_{3}$ are similar, according to the findings. Water has a viscosity of 47 nm above this region. The viscosity of Al${}_{2}$O${}_{3}$ nanofluid is significantly higher than that of water–36 nm Al${}_{2}$O${}_{3}$ nanofluid. As nanoparticles, Al${}_{2}$O${}_{3}$ and TiO${}_{2}$ were employed, using thermal oil as the base fluid. The needed amount of nanoparticles and base fluid were mixed. The diameter of alumina Al${}_{2}$O${}_{3}$ nanoparticles in spherical shape ranged from 5 nm to 250 nm, with a mean diameter of 47 nm according to the manufacturer (Sigma-Aldrich Co., Burlington, MA, USA) (see [30]).

This means that nanoparticles with a higher viscosity have better thermal, electrical, chemical, mechanical, magnetic, and optical capabilities. The viscosity of Al${}_{2}$O${}_{3}$ nanofluid is significantly higher than water–36 nm. Here, the particle volume fraction is more significant than 4%, why is why we are solely interested in studying 47 nm alumina particles. Additionally, Al${}_{2}$O${}_{3}$ and TiO${}_{2}$ were employed as nanoparticles, with thermal oil serving as the base fluid. The needed amount of nanoparticles and base fluid were mixed. The diameter of alumina Al${}_{2}$O${}_{3}$ nanoparticles in spherical shape ranged from 5 nm to 250 nm, with a mean diameter of 47 nm, according to the manufacturer (Sigma-Aldrich Co.) (see [30]). This is also the reason that we are talking only about the 47 nm alumina nanoparticles in our study.

The method we used in this paper and all the results obtained are given in this section. All 1001 points were obtained using the “NDSolve” command in Mathematica using the Runge-Kutta order-4 method. We set the step size 0.004 for receiving the 1001 points between 0 and 4. Then, we used those data set points in the neural toolbox using Matlab and suggest the LMB-NN method for the best result.

**Figure 4:**
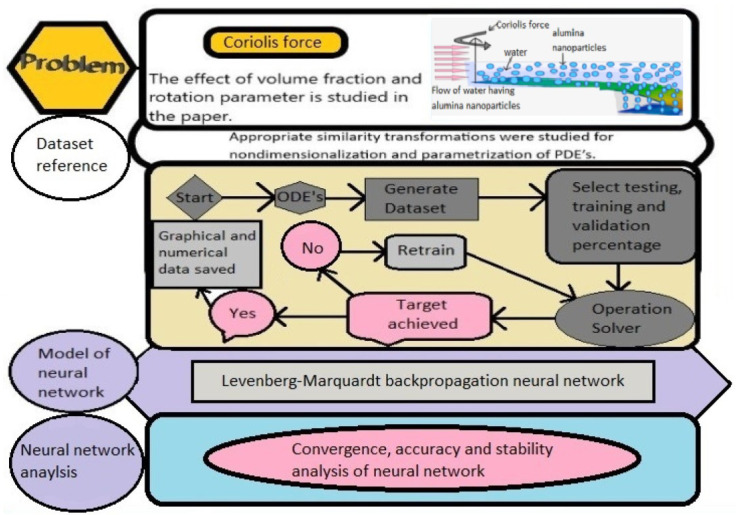
Flow chart of the proposed methodology.

Figure 4 shows us the basic working phenomenon of a neural network that how ANN works. We see that the neural network consists of inputs and the weights assigned to these inputs, then the hidden neuron, activation function, and, lastly, the outputs.

### Tabular and Statistical Analysis

Two scenarios are discussed in this paper; see Table 1. In scenario number 1, we change the value of rotation parameter K (0.1, 0.2, 0.3) and discuss the behavior of the solution of the system of ODEs and train a neural network for each case individually. Similarly, in scenario number 2, we change the value of volume fraction ϕ(0.1, 0.2, 0.3) and observe the behavior of the solution of the given system of ODEs and then train the neural network for each case and compare the results with RK4 data obtained by using the NDSolve command in Mathematica. We discussed two effects: the rotation parameter K and the volume fraction ϕ on the water containing 47 nm alumina nanoparticles. In Algorithm 1, all the procedures of the LMBNN are given in detail.

In Table 2 we present the numerical results obtained from the solution of LMB-NN. We discuss all the cases of both scenarios and observe the time taken to get the mean square error of the training, testing, and validation samples. Also shown are the performance, gradient, Mu values, and the epoch at which we got the good values. Table 3, Table 4, Table 5 and Table 6 show the comparison of RK4 and LMB-NN in all the three cases of scenario 1 in the solution of all the ODEs i.e., F, H, Θ, and Φ, respectively. As mentioned above, the first column in all the tables present the input value. The second column shows the results obtained by RK4, while the third column shows the results obtained through LMB-NN when K = 0.1. Similarly, the fourth and sixth columns represent the values of the results of RK4, while the fifth and seventh columns show the importance of LMB-NN when the value of K = 0.2 and K = 0.3, respectively. We can observe the data set of the methods, and it is clear that there is a tiny error between the data set of both approaches.
**Algorithm 1All the process of LMB-NN is given in the pseudocode****Starting of LMB-NN****Step 1: Construction**Construct input and data set**Step 2: Selection of Data**Target data and input data is chosen in non-linear form i.e., matrix form.**Step 3: Startup**Startup the ratio of Neuron numbers, testing, validation and training.▸ 90 Percent is for training▸ 5 percent is for validation▸ 5 percent is for testing▸ Number of hidden neurons is 40▸ Number of hidden layers is 4**Step 4: Weights for training**The selected data is trained from the activation function in LMB-NN**Step 5: Stopping criteria**Step 4 will stop automatically if the following conditions are satisfied.⋇ Reaching Mu to the maximum value⋇ Performance value reaches to minimum⋇ Maximum number of epoch achieved⋇ Performance of validation is less than maximum fail⋇ Gradient of performance less than minimum gradientTesting data help us determine that the network is generalized. If the outputs are good and useful forward to step 7, and if the outputs are not desirable, retrain the network.**Step 6: Retraining**For retraining the hidden neurons, the ratio of testing, training, and validation is changed. Then move again to step 4 and do the same procedure.**Phase 7: Output saving**The process is ended by saving the output simulation of data statistically as well as numerically.**Ending of LMB-NN**

**Figure 5:**
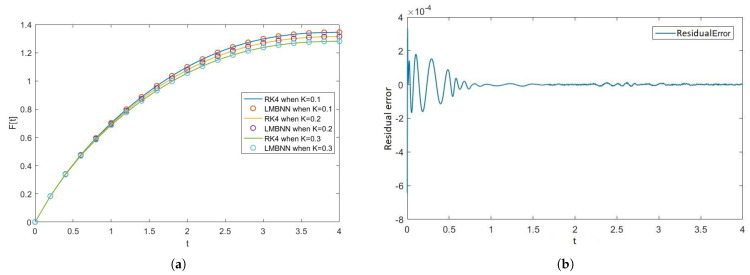

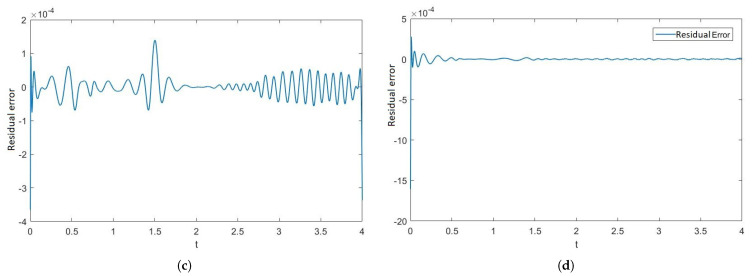
Graphical representation of F and the residual error graphs when rotation parameter K varies. (**a**) Graphical illustration of F when rotation parameter K varies. (**b**) Error in F when K = 0.1. (**c**) Error in F when K = 0.2. (**d**) Error in F when K = 0.3.

**Figure 6:**
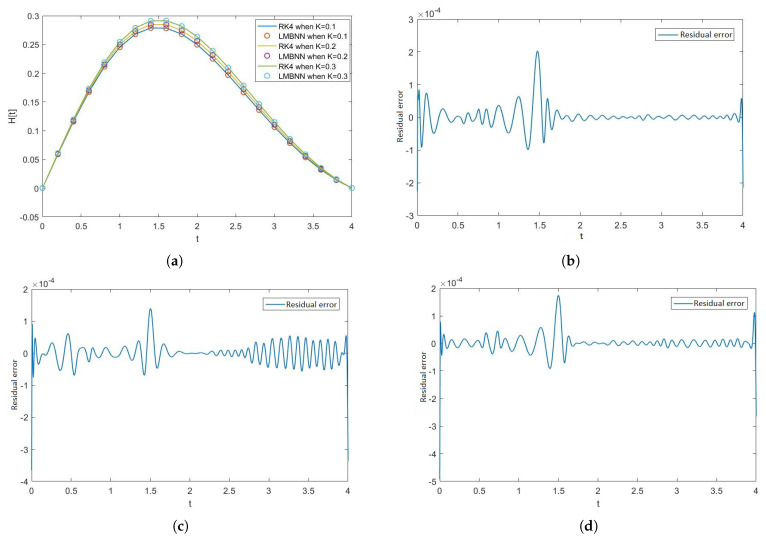
Graphical illustration of the effect of rotation parameter K on H and graphical illustration of error in the output data obtained through RK4 and LMBNN of H. (**a**) Graphs of H when K varies. (**b**) Error in H when K = 0.1. (**c**) Error in H when K = 0.2. (**d**) Error in H when K = 0.3.

**Figure 7:**
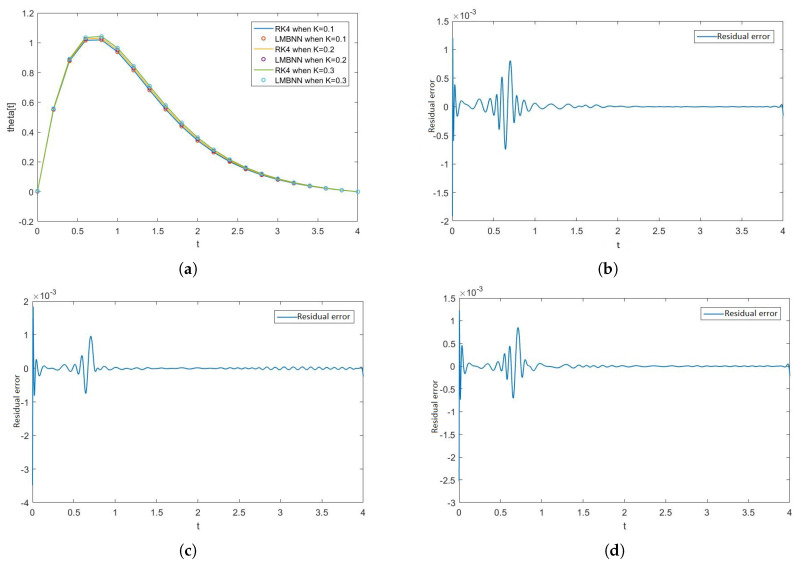
Figures of Θ and error graphs in the solution obtained from RK4 and LMBNN when K varies. (**a**) Graph of Θ when K varies. (**b**) Error in Θ K = 0.1. (**c**) Error in Θ K = 0.2. (**d**) Error in Θ K = 0.3.

**Figure 8:**
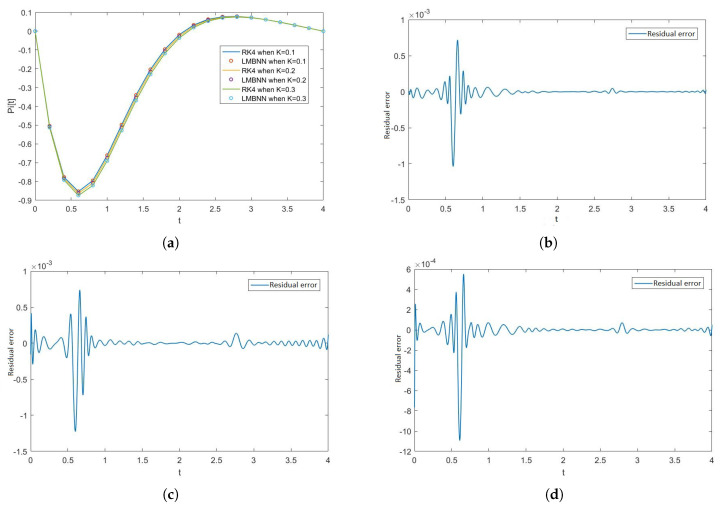
Effect of rotation parameter K on Φ and the graphs of error in numerical outputs of RK4 and LMBNN. (**a**) Numerical solutions of Φ when K varies. (**b**) Error in Φ when K = 0.1. (**c**) Error in Φ when K = 0.2. (**d**) Error in Φ when K = 0.3.

Figure 5, Figure 6, Figure 7 and Figure 8 show the solution and absolute error graphs of the variation of rotation parameter K of F, H, Θ, and Φ, respectively. In these graphs, we see for the given system of equations of ODEs, when we change the value of rotation parameter K, how the solution of the system of ODEs changes. The error graph shows the error between the RK4 solution and ANN solutions. The error between both methods is minimal, and both methods’ graphs overlap. The error graphs show us the residual error in the solution of both methods.

Figure 5a shows the numerical solutions of F of all the cases of scenario 1, while Figure 5b–d show the residual errors of F in case 1, case 2, and case 3 of scenario 1, respectively in the solutions of RK4 and LMB-NN. Figure 6a shows the numerical solutions of H of all the cases of scenario 1, while Figure 6b–d show the residual errors of H in case 1, case 2, and case 3 of scenario 1, respectively in the solutions of RK4 and LMB-NN. Figure 7a shows the numerical solutions of Θ of all the cases of scenario 1, while Figure 7b–d show the residual errors of Θ in case 1, case 2, and case 3 of scenario 1, respectively in the solutions of RK4 and LMB-NN. Figure 8a shows the numerical solutions of Φ of all the cases of scenario 1, while Figure 8b–d show the residual errors of Φ case 1, case 2, and case 3 of scenario 1, respectively in the solutions of RK4 and LMB-NN.

Scenario 1 is the variation of rotation parameter (K). We change the value of K and apply ANN for every individual value of the rotation parameter (K). In each case, we obtain the mean square error graphs, training graphs, error histogram graphs, regression graphs, and fitness graphs of the system of ODEs. We note how the values of the above change when we change the rotation parameter (K). The effect of the variation of K can be observed clearly from the values shown in the graphs. We train a neural network for each case by using the “nftool” command in the Matlab environment, setting the input and output data for the network, and using the LMB-NN method to find the best solution for the present system of ODEs. In this problem, we have one input and four hidden output layers. Moreover, we used 40 hidden neurons for a good result.

**Figure 9:**
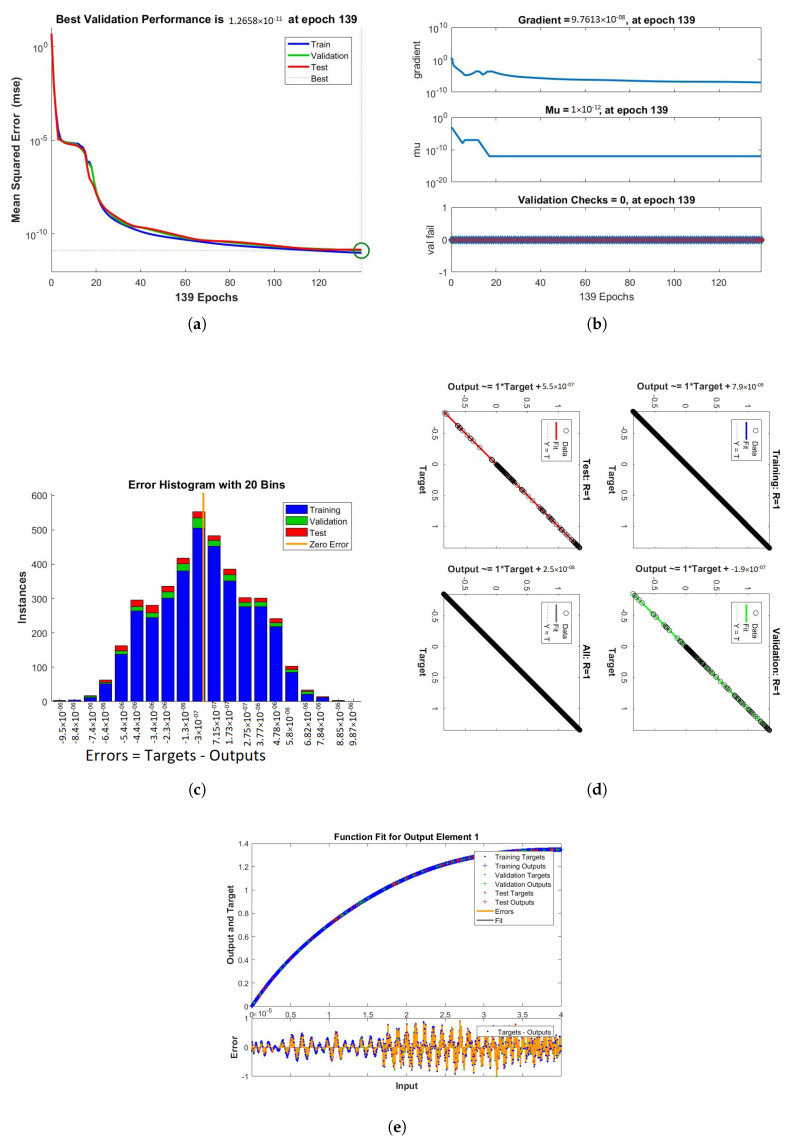
Performance, training, error histogram, regression, and fitness graphs of our system were obtained through LMBNN using the value of rotation parameter K = 0.1. (**a**) Performance. (**b**) Training. (**c**) Error Histogram. (**d**) Regression. (**e**) Fitness.

**Figure 10:**
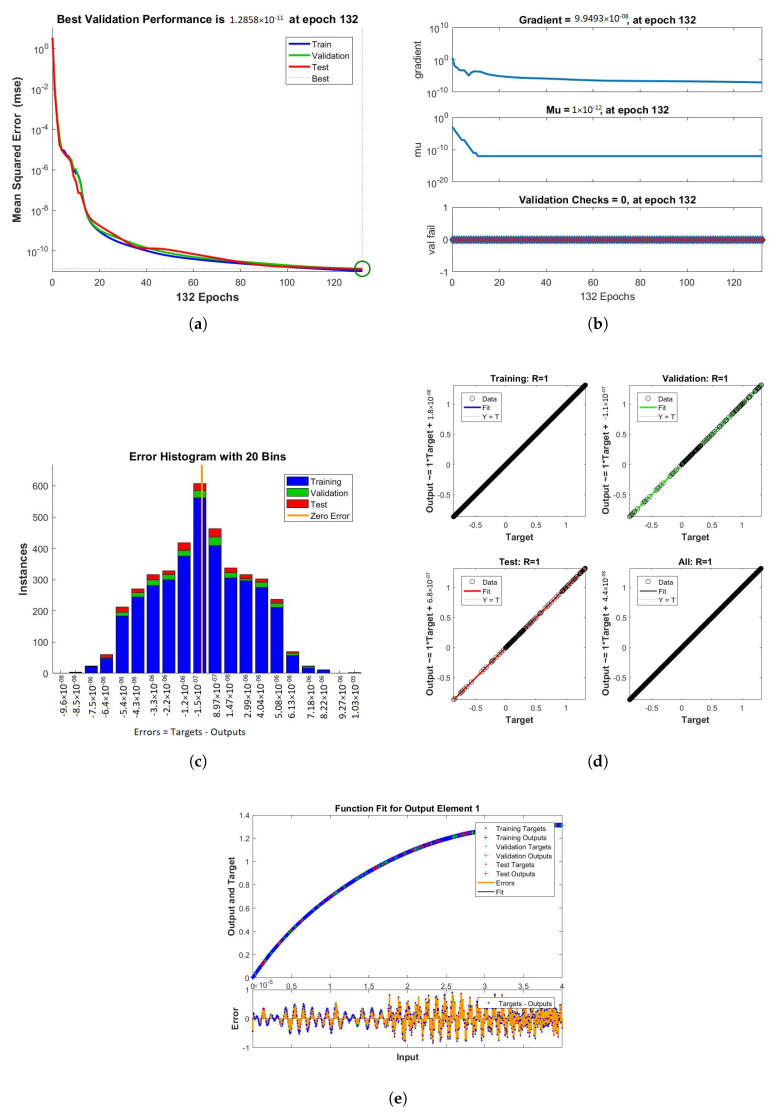
Figures of performance, training, error histogram, regression, and fitness of our system were obtained from LMBNN while using the value of rotation parameter K = 0.2. (**a**) Performance. (**b**) Training. (**c**) Error Histogram. (**d**) Regression. (**e**) Fitness.

**Figure 11:**
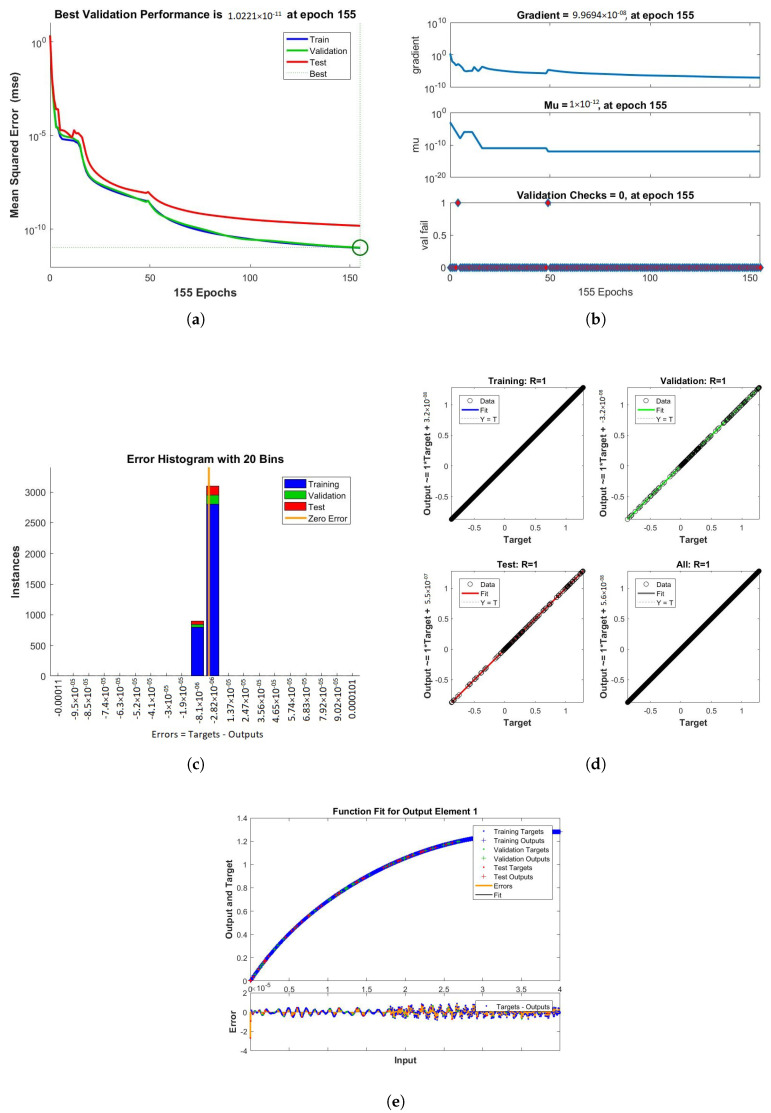
Performance, training, error histogram, regression, and fitness graphs of our system were obtained through LMBNN while using the value of rotation parameter K = 0.3. (**a**) Performance. (**b**) Training. (**c**) Error Histogram. (**d**) Regression. (**e**) Fitness.

The results obtained from LMB-NN of case 1, 2, and 3 of scenario 1 are shown in Figure 9, Figure 10 and Figure 11 respectively. These figures contain performance, training, error histogram, regression, and fitness graphs. Figure 9, Figure 10 and Figure 11 show the solutions obtained by artificial neural networks (ANNs). In these figures, we show the performance, training, error histogram, regression, and fitness graphs of the ANNs of all the cases of scenario 1. We use the input data of RK4 from Mathematica by using the “NDSolve” command. Figure 9a shows the performance of case 1 of scenario 1, in which we show the mean square error. Figure 9b shows the training samples of the ANNs of case 1 of scenario 1. Figure 9c shows the error histogram graph of case 1 of scenario 1. The regression of case 1 of scenario 1 is shown in Figure 9d and the fitness graphs of case 1 scenario 1 are shown in Figure 9e. Similarly, we show the performance, training, error histogram, regression, and the fitness figures in subfigures (a–e) of the remaining two cases of scenario 1 in Figure 10 and Figure 11, respectively.

When the value of MSE of a system is smaller, the system is stable. Among all the above cases discussed in the paper, the MSE of scenario 1 case 1 is smaller than all the other cases. The mean square error (MSE) of all the cases discussed in the paper ranges from $10^{-11}$ to $10^{-12}$. The Mu and gradient show a better rate of convergence. Auto-correlation shows the relationship between two variables. The $10^{-11}$ and $10^{-12}$ value of Mu shows us the better convergence. The histogram indicates the reliability of a technique, and the regression of all the above-discussed cases gives good results. The linear relationship between target data and output data are shown by regression. The data gives an accurate solution and is well trained investigated by fitness plots.

We obtained the RK4 outputs from the Mathematica window and then applied the LMBNN method in the neural network to get the outcomes of the technique. The errors between the results of both the methods are given in the Table 7, Table 8, Table 9 and Table 10. The residual error in the variation of the rotation parameter K in all the four ODEs can be easily observed from the tables. Column 1 of the above tables represents the input values, while columns 2, 3, and 4 illustrate the residual error present between the methods RK4 and LMB-NN obtained from Matlab when the rotation parameter K varies. The error in the tables is minimal, which shows that this technique has greater accuracy; moreover, the method is so simple and easy to implement on many problems of various fields and obtains fruitful results.

Table 11, Table 12, Table 13 and Table 14 show us the solution of all the ODEs (F, H,Θ,Φ) of the system of RK4 and LMB-NN methods of the variation of the volume fraction ϕ respectively. The comparison of RK4 and LMB-NN outputs are shown. The first column of Table 11, Table 12, Table 13 and Table 14 shows the input values. The second column shows the results obtained by the RK4 method, while the third column shows the results generated by LMB-NN at different input values. Similarly, the fourth and sixth columns represent the results generated by RK4, and the fifth and seventh columns of the tables show the results generated by LMB-NN at different input points, respectively. For each ODE, a separate table is given for comparison. All three cases of both scenarios are present in the tables.

**Figure 12:**
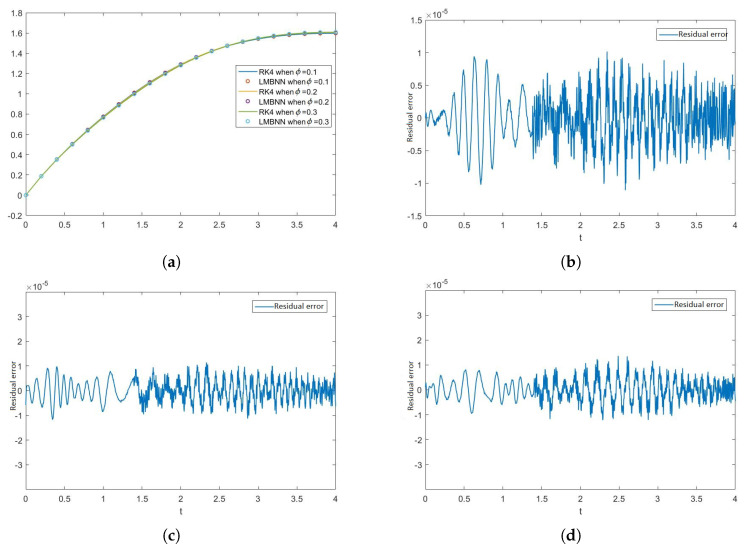
Graphical illustration of F and the residual error graphs in the outputs of RK4 and LMBNN when ϕ varies. (**a**) Numerical outputs of F when K varies. (**b**) Error in F when ϕ = 0.1. (**c**) Error in F when ϕ = 0.2. (**d**) Error in F when ϕ = 0.3.

**Figure 13:**
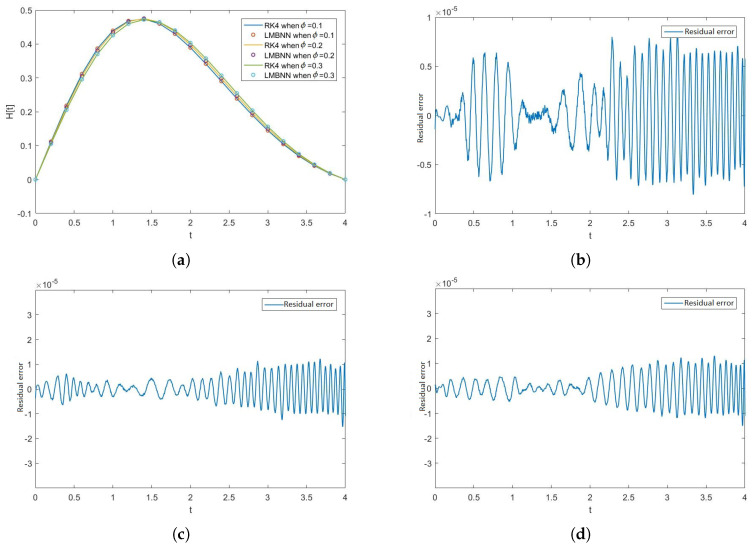
Effects of volume fraction ϕ on H and residual error graphs in the variance of ϕ. (**a**) Numerical outputs of H through RK4 and LMBNN for different values of ϕ. (**b**) Error in H when ϕ = 0.1. (**c**) Error in H when ϕ = 0.2. (**d**) Error in H when ϕ = 0.3.

**Figure 14:**
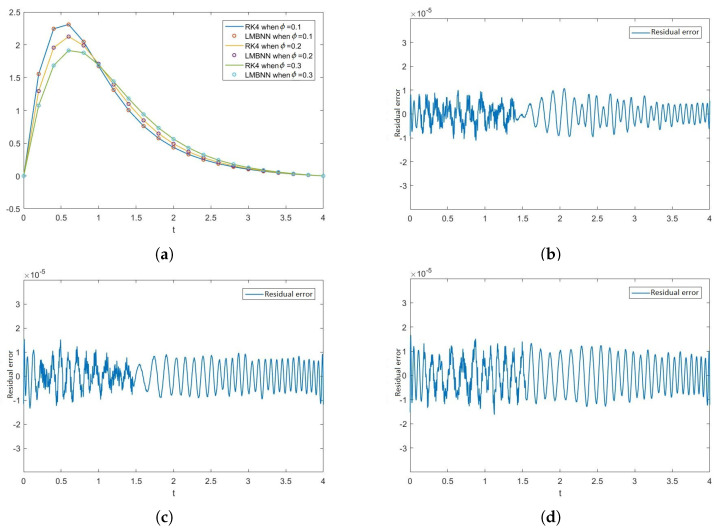
Graph of Θ when ϕ varies with the comparison of RK4 and LMBNN techniques and the graphs of the residual error between both the techniques. (**a**) Graphs of outputs of Θ obtained from RK4 and LMBNN technique when ϕ varies. (**b**) Error in Θ when ϕ = 0.1. (**c**) Error in Θ when ϕ = 0.2. (**d**) Error in Θ when ϕ = 0.3.

**Figure 15:**
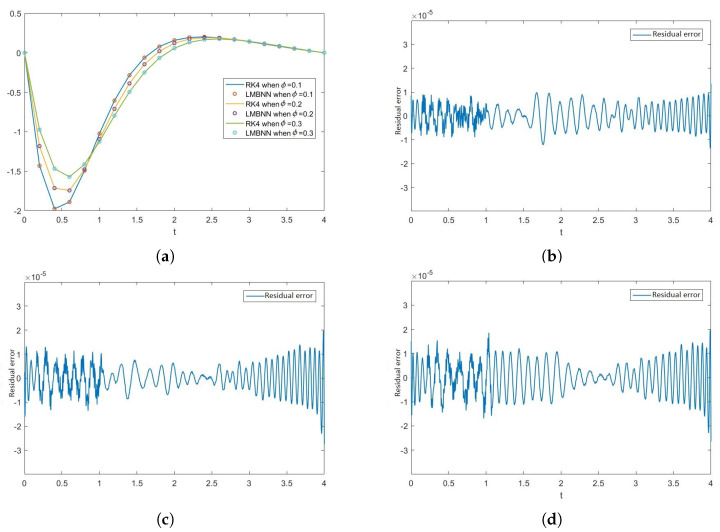
Graphs of Φ representing the outputs obtained from the RK4 and LMB-NN methods along with the graphs of the residual error between both the outputs. (**a**) Graphical representation of the outputs of Φ through the RK4 and LMBNN when ϕ varies. (**b**) Error graph of Φ when ϕ = 0.1. (**c**) Error graph of Φ when ϕ = 0.2. (**d**) Error graph of Φ when ϕ = 0.3.

Figure 12, Figure 13, Figure 14 and Figure 15 show the solution of the variation of volume fraction ϕ of all the ODEs along with the figures of absolute error. The effect of the volume fraction ϕ on F is given in Figure 12a. The errors in the variation of volume fraction ϕ (i.e., 0.1, 0.2, 0.3) are given in Figure 12b–d, respectively. Similarly, the effect of volume fraction ϕ on H is shown in Figure 13a and the absolute errors in the variation of volume fraction ϕ are shown in Figure 13b–d. In the same manner, the effect of ϕ and errors on the rest of the ODEs are shown in Figure 14 and Figure 15.

**Figure 16:**
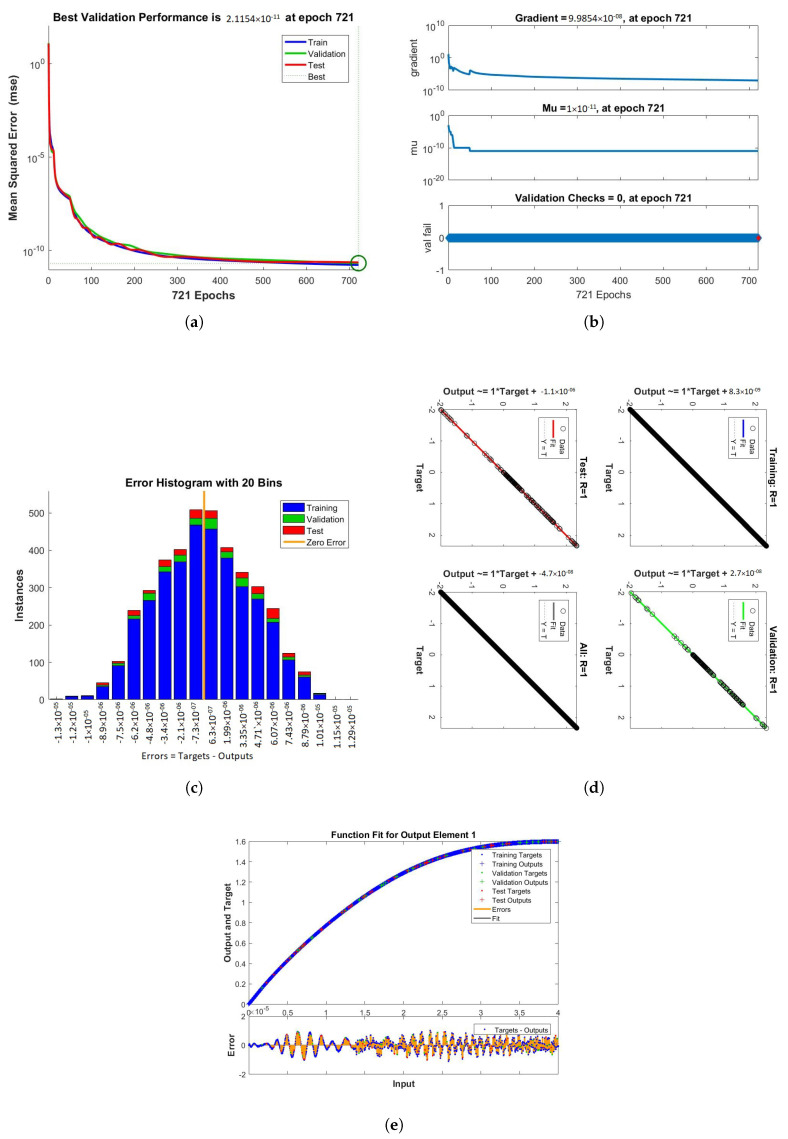
Figures showing the performance, training, error histogram, regression, and fitness graphs of the LMB-NN when value of the volume fraction ϕ = 0.1. (**a**) Performance. (**b**) Training. (**c**) Error Histogram. (**d**) Regression. (**e**) Fitness.

**Figure 17:**
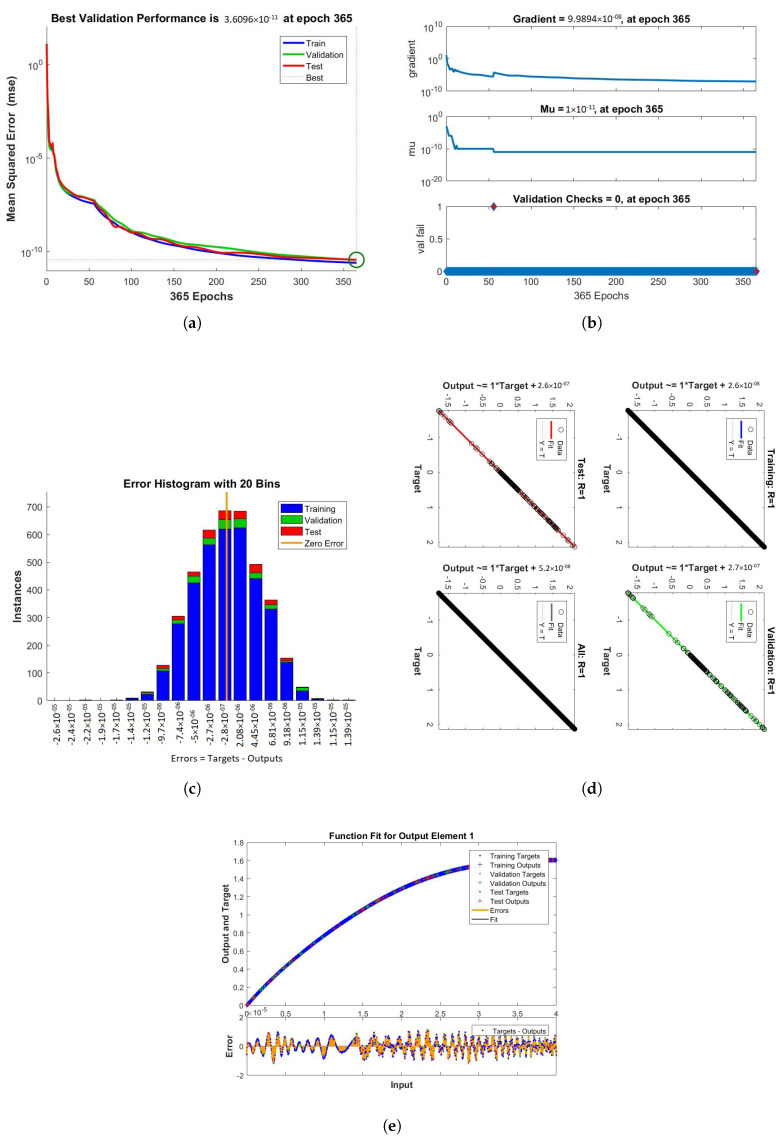
Figures showing the performance, training, error histogram, regression, and fitness graphs of the LMB-NN when value of the volume fraction ϕ = 0.2. (**a**) Performance. (**b**) Training. (**c**) Error Histogram. (**d**) Regression. (**e**) Fitness.

**Figure 18:**
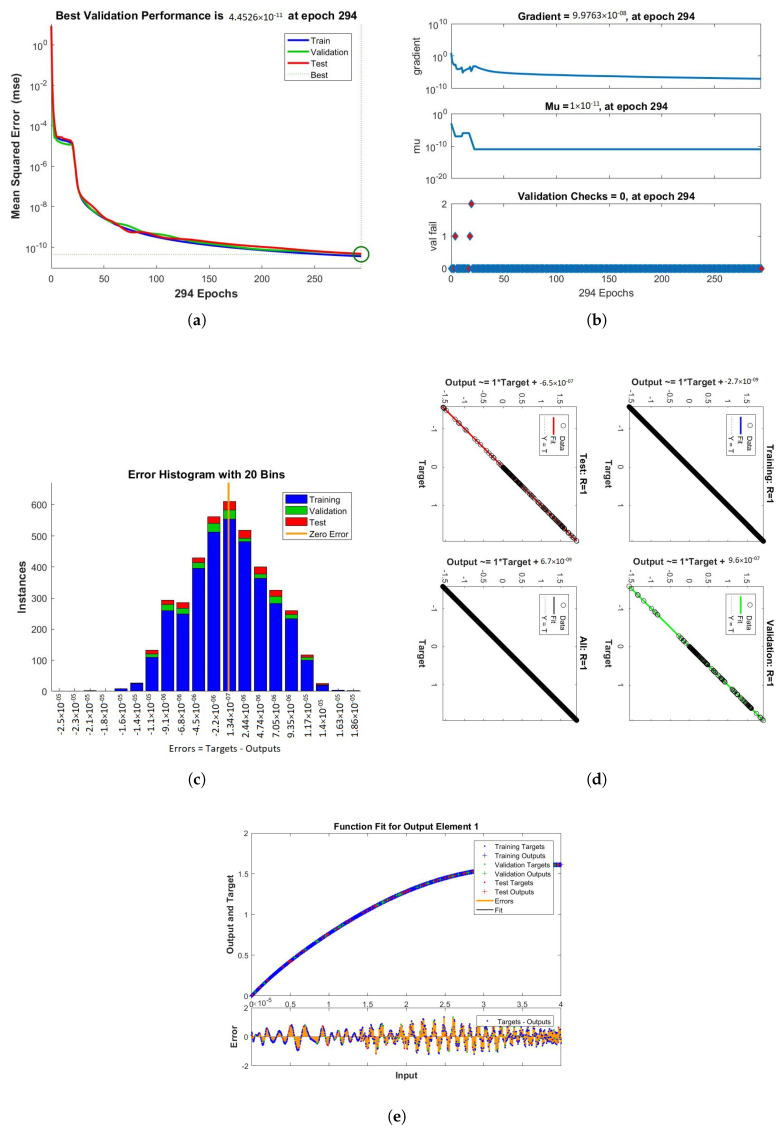
The figures clearly show the mean square error (mse), error histogram, gradient, Mu, validation checks, training, regression, and the epoch takes to reach the best results when ϕ = 0.3. (**a**) Performance. (**b**) Training. (**c**) Error Histogram. (**d**) Regression. (**e**) Fitness.

Figure 16, Figure 17 and Figure 18 show the solutions obtained by artificial neural networks (ANNs). In these graphs, we show the performance, training, error histogram, regression, and fitness graphs of the ANNs of all the cases of scenario 2. We use the input data of RK4 from Mathematica by using the “NDSolve” command. Figure 16a shows the performance of case 1 of scenario 2, in which we show the mean square error. Figure 16b shows the training samples of the ANNs of case 1 of scenario 2. Figure 16c shows the error histogram graph of case 1 of scenario 2. The regression of case of scenario 2 is shown in Figure 16d and the fitness graphs of case 1 scenario 2 are shown in Figure 16e. Similarly, we show the performance, training, error histogram, regression, and the fitness graphs in subfigures (a–e) of the remaining two cases of scenario 2 in Figure 17 and Figure 18, respectively.

In this paper, first, the outputs are obtained from Mathematica using the RK4 command, and then these outputs are used, and new outcomes are obtained through Matlab by using the “nftool” command for both the parameters. The error in both the outputs of all the four ODEs of the system when ϕ varies is given in Table 15, Table 16, Table 17 and Table 18. These tables show the residual errors in the outputs of both methods when different values of volume fraction ϕ are used. The first column of Table 15, Table 16, Table 17 and Table 18 represents the input values. The second column of the tables represents the residual error in both the methods when ϕ = 0.1. Similarly, the last two columns of the table represent the residual error in both methods when ϕ = 0.2 and ϕ = 0.3, respectively. The residual error is obtained from Matlab.

In our problem, we compare the solutions of artificial neural networks with the RK4 method, and it is clear from all the analyses that the solution of ANNs are entirely overlapping with the solutions of the RK4 method. Our method is best because we used the ANNs method having four hidden output layers, and the result is good to fast and accurate. Another thing is that our method is a computer-based method and an advanced method in which the chances of mistakes in the calculation are about negligible, and we can completely trust the results and require less time, and provide good results. The results of both scenarios’ cases are accurate, and significantly fewer errors mean that this method is very consistent. Moreover, the method is less time-consuming. This method may be used for various problems in the various fields to obtain accurate, fruitful, and valuable results, such as in the water present over a rotating surface in medicine, biodiesel, and maintaining cooling.

## 5. Conclusions

This study considers the industrial uses of nanofluids, knowing the effects of different parameters on the fluid, and how other parameters affect the heat transfer rate and the mass transfer rate in the flow of a nanofluid. The uses of 47 nm nanoparticles in the water present over a rotating surface can include medicine, biodiesel, maintaining cooling in different industries, etc. Variations of these nanoparticles still need to be investigated. The LMB-NN is a machine learning-based technique that is fast converging and accurate. All the results given by this method for all cases of both scenarios have minimal residual errors, which means that this method is consistent and reliable.

We have calculated solutions of problems under consideration with less mean squared errors (MSE); thus, it is established that our methodology is stable. In all test cases, which we have discussed in the paper, the MSE of scenario 1 case 1 is smaller than all the other cases. The mean square error (MSE) of all the cases discussed in the paper ranges from $10^{-11}$ to $10^{-12}$. The Mu and gradient show a better rate of convergence. Auto-correlation shows the relationship between two variables and the $10^{-11}$ to $10^{-12}$ value of Mu shows us the better convergence performance of our proposed strategy. The reliability of our technique is indicated by the histogram and regression plots, which show promising results in all scenarios. The linear relationship between target data and output data showed by regression plots is a further strength of our soft computing technique. Our solution technique gives an accurate solution, and fitness plots are presented for better clarity of our claims.

We have discussed variations in two parameters i.e., rotation parameter K and volume fraction ϕ, and compared our results with RK4. Our solutions are in strong agreement with the reference points. Our technique helps solve problems with unknown landscapes and is a robust procedure. From the outputs of our simulations, we see that it is very straightforward, easy, and simple to implement, and the method is computer-based and the outputs are matching with the reference inputs. The mean square error in the outputs of this method is very small; see Table 2. The mean square error range in this paper is $10^{-11}$ to $10^{-12}$. In the future, the new variants of the artificial intelligent networks based on the integrated intelligence will be used for many problems in different fields to obtain highly accurate and good results.

## Figures and Tables

**Table 1:** All the cases and scenarios discussed in the paper.

Senarios	Cases	Parameters
S1	C 1	K = 0.1
	C 2	K = 0.2
	C 3	K = 0.3
S2	C 1	ϕ = 0.1
	C 2	ϕ = 0.2
	C 3	ϕ = 0.3

**Table 2:** Numerical results of ANN of different cases of the rotation parameter K and the volume fraction ϕ showing the time taken, training, validation, testing, mean square error, gradient, and epoch to reach the best solution.

Scenario	Cases	Time	MSE			Performance	Gradiant	Mu	Epoch
			Training	Validation	Testing				
K	K = 0.1	17	9.57 $\times \;10^{-12}$	1.27 $\times \;10^{-11}$	1.43 $\times \;10^{-11}$	9.57 $\times \;10^{-12}$	9.76 $\times \;10^{-8}$	1.00 $\times \;10^{-12}$	139
	K = 0.2	11	1.00 $\times \;10^{-11}$	1.29 $\times \;10^{-11}$	1.22 $\times \;10^{-11}$	1.00 $\times \;10^{-11}$	9.95 $\times \;10^{-8}$	1.00 $\times \;10^{-12}$	132
	K = 0.3	14	9.92 $\times \;10^{-12}$	1.02 $\times \;10^{-11}$	1.51 $\times \;10^{-10}$	9.92 $\times \;10^{-12}$	9.97 $\times \;10^{-8}$	1.00 $\times \;10^{-12}$	155
ϕ	ϕ = 0.1	19	1.69 $\times \;10^{-11}$	2.12 $\times \;10^{-11}$	2.34 $\times \;10^{-11}$	1.69 $\times \;10^{-11}$	9.99 $\times \;10^{-8}$	1.00 $\times \;10^{-11}$	721
	ϕ = 0.2	10	2.50 $\times \;10^{-11}$	3.61 $\times \;10^{-11}$	3.57 $\times \;10^{-11}$	2.50 $\times \;10^{-11}$	9.99 $\times \;10^{-8}$	1.00 $\times \;10^{-11}$	365
	ϕ = 0.3	15	3.56 $\times \;10^{-11}$	4.45 $\times \;10^{-11}$	4.73 $\times \;10^{-11}$	3.56 $\times \;10^{-11}$	9.98 $\times \;10^{-8}$	1.00 $\times \;10^{-11}$	294

**Table 3:** Different values of F were obtained by RK4 and LMBNN methods when rotation parameter K varies (from 0.1–0.3).

F[t]						
**t**	**RK4**	**LMBNN**	**RK4**	**LMBNN**	**RK4**	**LMBNN**
	**K = 0.1**	**K = 0.1**	**K = 0.2**	**K = 0.2**	**K = 0.3**	**K = 0.3**
t = 0.00	0	0.000643	0	0.00104	0	0.001605
t = 0.2	0.183794	0.183933	0.183457	0.183548	0.183098	0.18311
t = 0.4	0.340317	0.340433	0.338988	0.339063	0.337568	0.337585
t = 0.6	0.47599	0.475981	0.473062	0.473039	0.469936	0.46993
t = 0.8	0.595633	0.595629	0.590591	0.590593	0.5852	0.5852
t = 1.00	0.702653	0.702656	0.695096	0.695082	0.687008	0.687014
t = 1.2	0.799272	0.799268	0.788941	0.788911	0.777864	0.777864
t = 1.4	0.886799	0.886807	0.873574	0.873574	0.859372	0.859354
t = 1.6	0.965899	0.965902	0.949798	0.94979	0.932478	0.932481
t = 1.8	1.03685	1.036848	1.01801	1.018005	0.997707	0.997713
t = 2.0	1.09974	1.09974	1.07839	1.078395	1.05534	1.055339
t = 2.2	1.15462	1.15462	1.13106	1.131062	1.10558	1.105586
t = 2.4	1.20162	1.201611	1.17618	1.17619	1.14862	1.148618
t = 2.6	1.24095	1.240945	1.21397	1.213969	1.1847	1.184696
t = 2.8	1.27298	1.272983	1.2448	1.244791	1.21418	1.214175
t = 3.0	1.2982	1.298199	1.26912	1.269101	1.23748	1.237475
t = 3.2	1.31721	1.317208	1.28749	1.287465	1.25513	1.255136
t = 3.4	1.33067	1.330676	1.30054	1.300527	1.2677	1.267698
t = 3.6	1.33933	1.339334	1.30896	1.308952	1.27584	1.275842
t = 3.8	1.34396	1.343964	1.31348	1.313467	1.28022	1.280214
t = 4.0	1.34533	1.34533	1.31482	1.314816	1.28152	1.281517

**Table 4:** The values of H by RK4 and LMB-NN methods at 21 different points when rotation parameter K varies.

H[t]						
**t**	**RK4**	**LMBNN**	**RK4**	**LMBNN**	**RK4**	**LMBNN**
	**K = 0.1**	**K = 0.1**	**K = 0.2**	**K = 0.2**	**K = 0.3**	**K = 0.3**
t = 0.00	0	0.000226	0	0.000365	0	0.000492
t = 0.2	0.059089	0.059138	0.059955	0.059955	0.060912	0.060922
t = 0.4	0.115841	0.115854	0.117584	0.117603	0.119511	0.119524
t = 0.6	0.167563	0.167562	0.170189	0.170177	0.173097	0.173067
t = 0.8	0.211534	0.211556	0.215034	0.215033	0.218911	0.218908
t = 1.00	0.245502	0.245465	0.249835	0.24984	0.25464	0.254616
t = 1.2	0.268067	0.268037	0.273155	0.273139	0.278804	0.278815
t = 1.4	0.278841	0.278868	0.284565	0.284601	0.290927	0.291016
t = 1.6	0.278401	0.278343	0.2846	0.284639	0.291501	0.291523
t = 1.8	0.268083	0.268085	0.274565	0.274572	0.281793	0.281789
t = 2.0	0.249718	0.249713	0.256271	0.256271	0.263593	0.263593
t = 2.2	0.225363	0.22537	0.231769	0.231772	0.238943	0.238933
t = 2.4	0.197077	0.197074	0.203131	0.203123	0.209927	0.209928
t = 2.6	0.166765	0.166766	0.172286	0.17229	0.178499	0.178504
t = 2.8	0.136078	0.136078	0.140918	0.140935	0.14638	0.146388
t = 3.0	0.106366	0.106367	0.11042	0.110453	0.115009	0.11502
t = 3.2	0.078679	0.078683	0.081886	0.081927	0.085526	0.085514
t = 3.4	0.053782	0.053776	0.056122	0.056153	0.058787	0.058794
t = 3.6	0.032193	0.032194	0.033687	0.033728	0.035394	0.035389
t = 3.8	0.014219	0.014212	0.014923	0.014967	0.015731	0.01574
t = 4.0	−6.71 $\times \;10^{-16}$	0.000214	−7.27 $\times \;10^{-16}$	0.000337	−9.45 $\times \;10^{-16}$	0.000264

**Table 5:** Comparison of RK4 and LM-BNN in Θ using different values of rotation parameter K.

Θ[t]						
**t**	**RK4**	**LMBNN**	**RK4**	**LMBNN**	**RK4**	**LMBNN**
	**K = 0.1**	**K = 0.1**	**K = 0.2**	**K = 0.2**	**K = 0.3**	**K = 0.3**
t = 0.00	0	0.001915	0	0.003481	0	0.005383
t = 0.2	0.551084	0.551072	0.554331	0.55434	0.55794	0.558028
t = 0.4	0.877823	0.877917	0.884118	0.884022	0.891115	0.891085
t = 0.6	1.0163	1.015775	1.02526	1.024887	1.03523	1.035112
t = 0.8	1.019	1.019042	1.03009	1.030103	1.04243	1.042399
t = 1.00	0.938167	0.938105	0.950697	0.95067	0.964648	0.964616
t = 1.2	0.816022	0.815974	0.829212	0.829239	0.843931	0.843901
t = 1.4	0.681857	0.68181	0.694948	0.694927	0.7096	0.70959
t = 1.6	0.553337	0.553312	0.565682	0.565687	0.579554	0.579547
t = 1.8	0.439393	0.439398	0.450525	0.450508	0.463092	0.463091
t = 2.0	0.343181	0.343176	0.352831	0.352816	0.363781	0.363787
t = 2.2	0.264487	0.26448	0.272565	0.272573	0.281778	0.281775
t = 2.4	0.201418	0.201416	0.207969	0.207953	0.215479	0.21548
t = 2.6	0.151473	0.151472	0.15663	0.156636	0.162571	0.162578
t = 2.8	0.112146	0.112145	0.116086	0.116104	0.120645	0.12065
t = 3.0	0.081212	0.081211	0.084123	0.084154	0.087504	0.087512
t = 3.2	0.056829	0.056828	0.058889	0.058923	0.061289	0.061276
t = 3.4	0.037529	0.037526	0.038896	0.038919	0.040493	0.040497
t = 3.6	0.022172	0.022172	0.022981	0.023008	0.023927	0.023923
t = 3.8	0.009885	0.00988	0.010244	0.010274	0.010666	0.010673
t = 4.0	−1.58 $\times \;10^{-16}$	0.000154	2.22 $\times \;10^{-16}$	0.00024	−1.39 $\times \;10^{-17}$	0.000185

**Table 6:** Different outputs of Φ using different values of rotation parameter K by solving on RK4 and LMBNN.

Φ[t]						
**t**	**RK4**	**LMBNN**	**RK4**	**LMBNN**	**RK4**	**LMBNN**
	**K = 0.1**	**K = 0.1**	**K = 0.2**	**K = 0.2**	**K = 0.3**	**K = 0.3**
t = 0.00	0	0.0000112	0	0.000153	0	0.000766
t = 0.2	−0.50429	−0.50421	−0.50798	−0.50803	−0.51209	−0.5121
t = 0.4	−0.77634	−0.77635	−0.78346	−0.78354	−0.7914	−0.79146
t = 0.6	−0.85358	−0.85254	−0.86364	−0.86241	−0.87485	−0.87385
t = 0.8	−0.79515	−0.79523	−0.80745	−0.80747	−0.82119	−0.82111
t = 1.00	−0.6608	−0.66077	−0.67447	−0.67443	−0.68974	−0.68981
t = 1.2	−0.49841	−0.49835	−0.51244	−0.51247	−0.52817	−0.52822
t = 1.4	−0.34007	−0.34004	−0.35351	−0.35349	−0.36865	−0.36868
t = 1.6	−0.20365	−0.20363	−0.21571	−0.21572	−0.2294	−0.22939
t = 1.8	−0.09637	−0.09637	−0.10655	−0.10655	−0.1182	−0.11819
t = 2.0	−0.01865	−0.01865	−0.02672	−0.02673	−0.03606	−0.03607
t = 2.2	0.032836	0.032835	0.026852	0.026835	0.019826	0.019831
t = 2.4	0.06294	0.062943	0.058835	0.05884	0.053918	0.053924
t = 2.6	0.076726	0.076731	0.074184	0.074157	0.071043	0.071049
t = 2.8	0.078741	0.078762	0.077402	0.077338	0.075649	0.075584
t = 3.0	0.072728	0.072724	0.072236	0.072262	0.071485	0.071493
t = 3.2	0.06158	0.061583	0.061619	0.061636	0.061513	0.061517
t = 3.4	0.047439	0.047436	0.047741	0.047752	0.047978	0.047979
t = 3.6	0.031827	0.031828	0.032178	0.032207	0.03251	0.032504
t = 3.8	0.015786	0.015784	0.016021	0.016063	0.016258	0.016264
t = 4.0	1.54 $\times \;10^{-16}$	−2.74 $\times \;10^{-5}$	3.57 $\times \;10^{-16}$	−1.2 $\times \;10^{-4}$	6.26 $\times \;10^{-16}$	−4.94 $\times \;10^{-5}$

**Table 7:** Residual error in F when rotation parameter K varies.

Residual Error in F			
**t**	**Residual Error When K = 0.1**	**Residual Error When K = 0.2**	**Residual Error When K = 0.3**
0	−6.43 $\times \;10^{-4}$	−1.04 $\times \;10^{-3}$	−1.60 $\times \;10^{-3}$
0.2	−1.39 $\times \;10^{-4}$	−9.10 $\times \;10^{-5}$	−1.20 $\times \;10^{-5}$
0.4	−1.16 $\times \;10^{-4}$	−7.53 $\times \;10^{-5}$	−1.70 $\times \;10^{-5}$
0.6	9.43 $\times \;10^{-6}$	2.34 $\times \;10^{-5}$	6.19 $\times \;10^{-6}$
0.8	4.25 $\times \;10^{-6}$	−1.68 $\times \;10^{-6}$	4.68 $\times \;10^{-7}$
1	−2.63 $\times \;10^{-6}$	1.36 $\times \;10^{-5}$	−5.88 $\times \;10^{-6}$
1.2	4.02 $\times \;10^{-6}$	3.05 $\times \;10^{-5}$	−2.81 $\times \;10^{-7}$
1.4	−7.89 $\times \;10^{-6}$	−2.06 $\times \;10^{-7}$	1.84 $\times \;10^{-5}$
1.6	−3.00 $\times \;10^{-6}$	7.54 $\times \;10^{-6}$	−3.28 $\times \;10^{-6}$
1.8	1.94 $\times \;10^{-6}$	4.84 $\times \;10^{-6}$	−5.69 $\times \;10^{-6}$
2	−2.32 $\times \;10^{-7}$	−5.38 $\times \;10^{-6}$	1.34 $\times \;10^{-6}$
2.2	−2.44 $\times \;10^{-8}$	−1.55 $\times \;10^{-6}$	−6.47 $\times \;10^{-6}$
2.4	8.79 $\times \;10^{-6}$	−1.00 $\times \;10^{-5}$	2.30 $\times \;10^{-6}$
2.6	5.18 $\times \;10^{-6}$	1.21 $\times \;10^{-6}$	3.56 $\times \;10^{-6}$
2.8	−2.60 $\times \;10^{-6}$	9.06 $\times \;10^{-6}$	5.21 $\times \;10^{-6}$
3	1.43 $\times \;10^{-6}$	1.87 $\times \;10^{-5}$	4.93 $\times \;10^{-6}$
3.2	2.30 $\times \;10^{-6}$	2.50 $\times \;10^{-5}$	−5.97 $\times \;10^{-6}$
3.4	−5.54 $\times \;10^{-6}$	1.29 $\times \;10^{-5}$	2.36 $\times \;10^{-6}$
3.6	−4.40 $\times \;10^{-6}$	7.97 $\times \;10^{-6}$	−2.48 $\times \;10^{-6}$
3.8	−4.43 $\times \;10^{-6}$	1.32 $\times \;10^{-5}$	5.80 $\times \;10^{-6}$
4	2.60 $\times \;10^{-7}$	3.90 $\times \;10^{-6}$	2.73 $\times \;10^{-6}$

**Table 8:** Residual error in H between the Mathematica and neural outputs.

Residual Error in H			
**t**	**Residual Error When K = 0.1**	**Residual Error When K = 0.2**	**Residual Error When K = 0.3**
0	−2.26 $\times \;10^{-4}$	−3.65 $\times \;10^{-4}$	−4.92 $\times \;10^{-4}$
0.2	−4.90 $\times \;10^{-5}$	1.90 $\times \;10^{-7}$	−9.72 $\times \;10^{-6}$
0.4	−1.28 $\times \;10^{-5}$	−1.91 $\times \;10^{-5}$	−1.25 $\times \;10^{-5}$
0.6	1.46 $\times \;10^{-6}$	1.15 $\times \;10^{-5}$	2.98 $\times \;10^{-5}$
0.8	−2.17 $\times \;10^{-5}$	1.22 $\times \;10^{-6}$	3.04 $\times \;10^{-6}$
1	3.68 $\times \;10^{-5}$	−5.03 $\times \;10^{-6}$	2.39 $\times \;10^{-5}$
1.2	2.96 $\times \;10^{-5}$	1.63 $\times \;10^{-5}$	−1.13$\times 10^{-5}$
1.4	−2.69 $\times \;10^{-5}$	−3.57$\times 10^{-5}$	−8.88 $\times \;10^{-5}$
1.6	5.77 $\times \;10^{-5}$	−3.91 $\times \;10^{-5}$	−2.24 $\times \;10^{-5}$
1.8	−2.29 $\times \;10^{-6}$	−6.56 $\times \;10^{-6}$	3.60 $\times \;10^{-6}$
2	5.42 $\times \;10^{-6}$	−1.87 $\times \;10^{-7}$	2.34 $\times \;10^{-7}$
2.2	−6.81 $\times \;10^{-6}$	−2.91 $\times \;10^{-6}$	9.74 $\times \;10^{-6}$
2.4	3.14 $\times \;10^{-6}$	7.62 $\times \;10^{-6}$	−9.27 $\times \;10^{-7}$
2.6	−7.15 $\times \;10^{-7}$	−3.81 $\times \;10^{-6}$	−4.54 $\times \;10^{-6}$
2.8	−2.39 $\times \;10^{-7}$	−1.68 $\times \;10^{-5}$	−7.62 $\times \;10^{-6}$
3	−7.62 $\times \;10^{-7}$	−3.33 $\times \;10^{-5}$	−1.07 $\times \;10^{-5}$
3.2	−3.75 $\times \;10^{-6}$	−4.10 $\times \;10^{-5}$	1.21 $\times \;10^{-5}$
3.4	6.78 $\times \;10^{-6}$	−3.06 $\times \;10^{-5}$	−6.99 $\times \;10^{-6}$
3.6	−9.27 $\times \;10^{-7}$	−4.06 $\times \;10^{-5}$	5.52 $\times \;10^{-6}$
3.8	6.95 $\times \;10^{-6}$	−4.39 $\times \;10^{-5}$	−9.77 $\times \;10^{-6}$
4	−2.14 $\times \;10^{-4}$	−3.37 $\times \;10^{-4}$	−2.64 $\times \;10^{-4}$

**Table 9:** Residual error with the reference solution of RK4 in Θ. These residual errors are obtained from Matlab.

Residual Error in Θ			
**t**	**Residual Error When K = 0.1**	**Residual Error When K = 0.2**	**Residual Error When K = 0.3**
0	−1.92 $\times \;10^{-3}$	−3.48 $\times \;10^{-3}$	−5.38 $\times \;10^{-3}$
0.2	1.25 $\times \;10^{-5}$	−9.31 $\times \;10^{-6}$	−8.81 $\times \;10^{-5}$
0.4	−9.39 $\times \;10^{-5}$	9.61 $\times \;10^{-5}$	3.02 $\times \;10^{-5}$
0.6	5.25 $\times \;10^{-4}$	3.73 $\times \;10^{-4}$	1.18 $\times \;10^{-4}$
0.8	−4.17 $\times \;10^{-5}$	−1.34 $\times \;10^{-5}$	3.07 $\times \;10^{-5}$
1	6.16 $\times \;10^{-5}$	2.73 $\times \;10^{-5}$	3.24 $\times \;10^{-5}$
1.2	4.81 $\times \;10^{-5}$	−2.70 $\times \;10^{-5}$	3.02 $\times \;10^{-5}$
1.4	4.71 $\times \;10^{-5}$	2.07 $\times \;10^{-5}$	1.01 $\times \;10^{-5}$
1.6	2.50 $\times \;10^{-5}$	−5.03 $\times \;10^{-6}$	7.11 $\times \;10^{-6}$
1.8	−5.45 $\times \;10^{-6}$	1.70 $\times \;10^{-5}$	9.49 $\times \;10^{-7}$
2	5.23 $\times \;10^{-6}$	1.48 $\times \;10^{-5}$	−6.01 $\times \;10^{-6}$
2.2	7.39 $\times \;10^{-6}$	−7.77 $\times \;10^{-6}$	2.62 $\times \;10^{-6}$
2.4	2.17 $\times \;10^{-6}$	1.60 $\times \;10^{-5}$	−6.64 $\times \;10^{-7}$
2.6	1.14 $\times \;10^{-6}$	−6.22 $\times \;10^{-6}$	−7.10 $\times \;10^{-6}$
2.8	5.46 $\times \;10^{-7}$	−1.77 $\times \;10^{-5}$	−4.74 $\times \;10^{-6}$
3	5.71 $\times \;10^{-7}$	−3.08 $\times \;10^{-5}$	−8.59 $\times \;10^{-6}$
3.2	5.45 $\times \;10^{-7}$	−3.44 $\times \;10^{-5}$	1.25 $\times \;10^{-5}$
3.4	2.62 $\times \;10^{-6}$	−2.29 $\times \;10^{-5}$	−3.71 $\times \;10^{-6}$
3.6	−5.12 $\times \;10^{-7}$	−2.79 $\times \;10^{-5}$	4.18 $\times \;10^{-6}$
3.8	4.56 $\times \;10^{-6}$	−2.98 $\times \;10^{-5}$	−6.62 $\times \;10^{-6}$
4	−1.54 $\times \;10^{-4}$	−2.40 $\times \;10^{-4}$	−1.85 $\times \;10^{-4}$

**Table 10:** Residual error in RK4 and LMB-NN in Φ.

Residual Error in Φ			
**t**	**Residual Error When K = 0.1**	**Residual Error When K = 0.2**	**Residual Error When K = 0.3**
0	−1.12 $\times \;10^{-5}$	−1.53 $\times \;10^{-4}$	−7.66 $\times \;10^{-4}$
0.2	−7.59 $\times \;10^{-5}$	4.73 $\times \;10^{-5}$	6.38 $\times \;10^{-6}$
0.4	8.83 $\times \;10^{-6}$	7.49 $\times \;10^{-5}$	6.41 $\times \;10^{-5}$
0.6	−1.04 $\times \;10^{-3}$	−1.23 $\times \;10^{-3}$	−1.00 $\times \;10^{-3}$
0.8	8.72 $\times \;10^{-5}$	1.97 $\times \;10^{-5}$	−7.58 $\times \;10^{-5}$
1	−3.62 $\times \;10^{-5}$	−3.18 $\times \;10^{-5}$	6.76 $\times \;10^{-5}$
1.2	−5.75 $\times \;10^{-5}$	3.16 $\times \;10^{-5}$	5.20 $\times \;10^{-5}$
1.4	−3.38 $\times \;10^{-5}$	−1.60 $\times \;10^{-5}$	2.92 $\times \;10^{-5}$
1.6	−1.34 $\times \;10^{-5}$	6.64 $\times \;10^{-6}$	−7.06 $\times \;10^{-6}$
1.8	3.54 $\times \;10^{-6}$	−5.05 $\times \;10^{-6}$	−1.06 $\times \;10^{-5}$
2	−1.39 $\times \;10^{-7}$	8.68 $\times \;10^{-6}$	8.21 $\times \;10^{-6}$
2.2	8.88 $\times \;10^{-7}$	1.73 $\times \;10^{-5}$	−5.32 $\times \;10^{-6}$
2.4	−3.96 $\times \;10^{-6}$	−5.11 $\times \;10^{-6}$	−6.21 $\times \;10^{-6}$
2.6	−4.35 $\times \;10^{-6}$	2.76 $\times \;10^{-5}$	−5.77 $\times \;10^{-6}$
2.8	−2.01 $\times \;10^{-5}$	6.31 $\times \;10^{-5}$	6.54 $\times \;10^{-5}$
3	3.46 $\times \;10^{-6}$	−2.58 $\times \;10^{-5}$	−8.42 $\times \;10^{-6}$
3.2	−2.51 $\times \;10^{-6}$	−1.68 $\times \;10^{-5}$	−4.01 $\times \;10^{-6}$
3.4	3.31 $\times \;10^{-6}$	−1.12 $\times \;10^{-5}$	−1.36 $\times \;10^{-6}$
3.6	−6.46 $\times \;10^{-7}$	−2.97 $\times \;10^{-5}$	6.47 $\times \;10^{-6}$
3.8	1.78 $\times \;10^{-6}$	−4.25 $\times \;10^{-5}$	−5.95 $\times \;10^{-6}$
4	2.74 $\times \;10^{-5}$	1.19 $\times \;10^{-4}$	4.94 $\times \;10^{-5}$

**Table 11:** Numerical values of F obtained through RK4 and LMBNN using different values of volume fraction ϕ.

F[t]						
**t**	**RK4**	**LMBNN**	**RK4**	**LMBNN**	**RK4**	**LMBNN**
	**ϕ = 0.1**	**ϕ = 0.1**	**ϕ = 0.2**	**ϕ = 0.2**	**ϕ = 0.3**	**ϕ = 0.3**
t = 0.00	0	6.89 $\times \;10^{-8}$	0	5.75 $\times \;10^{-6}$	0	−1.04 $\times \;10^{-6}$
t = 0.2	0.187068	0.187068	0.186821	0.186827	0.186496	0.18649
t = 0.4	0.353267	0.353268	0.352319	0.352309	0.35106	0.35106
t = 0.6	0.504478	0.504477	0.502514	0.502511	0.499843	0.499852
t = 0.8	0.644506	0.644498	0.641436	0.641433	0.637104	0.637107
t = 1.00	0.77533	0.775332	0.771328	0.771336	0.765371	0.765375
t = 1.2	0.89754	0.897541	0.893	0.893004	0.885734	0.885733
t = 1.4	1.01085	1.010849	1.00629	1.006288	0.998239	0.998239
t = 1.6	1.11456	1.114557	1.11053	1.110528	1.10231	1.102315
t = 1.8	1.20794	1.207934	1.2049	1.204899	1.19712	1.197119
t = 2.0	1.29044	1.290435	1.28873	1.288732	1.28191	1.281915
t = 2.2	1.36182	1.361824	1.36163	1.361627	1.35612	1.356118
t = 2.4	1.4222	1.422204	1.42356	1.423569	1.41956	1.419565
t = 2.6	1.47198	1.471986	1.47482	1.474828	1.47236	1.472357
t = 2.8	1.51185	1.511851	1.516	1.516006	1.515	1.514998
t = 3.0	1.54268	1.54268	1.54793	1.547925	1.54818	1.548188
t = 3.2	1.56547	1.565475	1.57158	1.571576	1.57286	1.572856
t = 3.4	1.5813	1.581304	1.58803	1.588027	1.59006	1.590061
t = 3.6	1.59128	1.59128	1.5984	1.598403	1.60094	1.600943
t = 3.8	1.59649	1.596489	1.60382	1.603825	1.60663	1.606631
t = 4.0	1.598	1.598001	1.60539	1.605396	1.60828	1.608284

**Table 12:** Comparison of output data obtained through RK4 and LMBNN methods when volume fraction ϕ varies.

H[t]						
**t**	**RK4**	**LMBNN**	**RK4**	**LMBNN**	**RK4**	**LMBNN**
	**ϕ = 0.1**	**ϕ = 0.1**	**ϕ = 0.2**	**ϕ = 0.2**	**ϕ = 0.3**	**ϕ = 0.3**
t = 0.00	0	1.38 $\times \;10^{-6}$	0	3.57 $\times \;10^{-6}$	0	−1.06 $\times \;10^{-6}$
t = 0.2	0.112012	0.112013	0.109136	0.10914	0.105157	0.105154
t = 0.4	0.218049	0.218052	0.212854	0.212848	0.205469	0.205469
t = 0.6	0.311604	0.311604	0.305211	0.30521	0.295592	0.295595
t = 0.8	0.386718	0.386713	0.380604	0.380603	0.370367	0.370366
t = 1.00	0.439333	0.439334	0.434919	0.434922	0.425781	0.42578
t = 1.2	0.468008	0.468008	0.466286	0.466286	0.45967	0.459669
t = 1.4	0.473838	0.473838	0.475187	0.475189	0.471954	0.471955
t = 1.6	0.459837	0.459836	0.464052	0.464055	0.464411	0.464408
t = 1.8	0.430122	0.430124	0.436587	0.436588	0.44018	0.44018
t = 2.0	0.389172	0.389174	0.397067	0.397066	0.40315	0.403147
t = 2.2	0.341269	0.341269	0.349757	0.34976	0.357405	0.357411
t = 2.4	0.29016	0.290156	0.298509	0.298503	0.306799	0.306794
t = 2.6	0.238896	0.238896	0.246545	0.246538	0.254674	0.254683
t = 2.8	0.189807	0.189812	0.196384	0.196382	0.20374	0.203741
t = 3.0	0.144556	0.144562	0.149865	0.149873	0.156045	0.156043
t = 3.2	0.104236	0.104233	0.108226	0.10823	0.113022	0.113023
t = 3.4	0.069475	0.069478	0.072208	0.072211	0.075581	0.075591
t = 3.6	0.040543	0.040538	0.042162	0.042151	0.044203	0.044191
t = 3.8	0.017446	0.017453	0.018143	0.018138	0.01904	0.019047
t = 4.0	−2.14 $\times \;10^{-15}$	−5.78 $\times \;10^{-6}$	9.35 $\times \;10^{-16}$	1.09 $\times \;10^{-5}$	2.39 $\times \;10^{-15}$	1.10 $\times \;10^{-5}$

**Table 13:** Comparison of different values of Θ between RK4 and LMB-NN when ϕ varies.

Θ[t]						
**t**	**RK4**	**LMBNN**	**RK4**	**LMBNN**	**RK4**	**LMBNN**
	**ϕ = 0.1**	**ϕ = 0.1**	**ϕ = 0.2**	**ϕ = 0.2**	**ϕ = 0.3**	**ϕ = 0.3**
t = 0.00	0	9.21 $\times \;10^{-6}$	0	1.17 $\times \;10^{-5}$	0	1.52 $\times \;10^{-5}$
t = 0.2	1.55534	1.55534	1.29423	1.294232	1.07413	1.074122
t = 0.4	2.24418	2.244179	1.95594	1.955931	1.68327	1.683266
t = 0.6	2.30933	2.309327	2.12525	2.125243	1.91241	1.912411
t = 0.8	2.04656	2.046563	1.98927	1.989274	1.87847	1.878473
t = 1.00	1.67446	1.674458	1.70941	1.709413	1.69298	1.692973
t = 1.2	1.3112	1.311196	1.39302	1.393024	1.44156	1.441564
t = 1.4	1.00358	1.003578	1.09775	1.097751	1.17994	1.17994
t = 1.6	0.760452	0.760447	0.847338	0.847339	0.939035	0.939026
t = 1.8	0.574602	0.574605	0.646197	0.646202	0.732419	0.732429
t = 2.0	0.434326	0.434333	0.48954	0.489543	0.563026	0.563016
t = 2.2	0.328466	0.32846	0.369386	0.369392	0.428108	0.428115
t = 2.4	0.247991	0.247986	0.277626	0.277619	0.32249	0.322486
t = 2.6	0.186196	0.186196	0.207357	0.207353	0.240513	0.240525
t = 2.8	0.138229	0.138231	0.153163	0.15316	0.177007	0.177006
t = 3.0	0.100596	0.100601	0.110977	0.110983	0.127668	0.127665
t = 3.2	0.070804	0.070804	0.077828	0.077832	0.089114	0.089114
t = 3.4	0.047035	0.047036	0.051549	0.051551	0.058768	0.058775
t = 3.6	0.027938	0.027934	0.030551	0.030543	0.034697	0.034688
t = 3.8	0.012514	0.012518	0.01366	0.013656	0.015465	0.015471
t = 4.0	6.07 $\times \;10^{-17}$	−5.54 $\times \;10^{-6}$	4.81 $\times \;10^{-16}$	1.18 $\times \;10^{-5}$	5.64 $\times \;10^{-16}$	1.21 $\times \;10^{-5}$

**Table 14:** Numerical outputs of Φ at different values of ϕ using RK4 and LMBNN techniques.

Φ[t]						
**t**	**RK4**	**LMBNN**	**RK4**	**LMBNN**	**RK4**	**LMBNN**
	**ϕ = 0.1**	**ϕ = 0.1**	**ϕ = 0.2**	**ϕ = 0.2**	**ϕ = 0.3**	**ϕ = 0.3**
t = 0.00	0	−8.62 $\times \;10^{-6}$	0	−2.56 $\times \;10^{-6}$	0	−1.50 $\times \;10^{-5}$
t = 0.2	−1.43028	−1.43028	−1.18136	−1.18136	−0.97385	−0.97384
t = 0.4	−1.97489	−1.97489	−1.71308	−1.71309	−1.46774	−1.46773
t = 0.6	−1.88905	−1.88906	−1.74254	−1.74253	−1.57038	−1.57038
t = 0.8	−1.49282	−1.49283	−1.47602	−1.47602	−1.41311	−1.41312
t = 1.00	−1.02319	−1.02319	−1.09275	−1.09275	−1.12306	−1.12306
t = 1.2	−0.60554	−0.60553	−0.71023	−0.71023	−0.79701	−0.79701
t = 1.4	−0.28453	−0.28453	−0.38801	−0.388	−0.49553	−0.49553
t = 1.6	−0.06174	−0.06174	−0.14572	−0.14572	−0.24874	−0.24873
t = 1.8	0.079054	0.079055	0.019518	0.019516	−0.06549	−0.0655
t = 2.0	0.158181	0.158177	0.120693	0.120688	0.057984	0.057989
t = 2.2	0.193992	0.193999	0.173363	0.173363	0.131567	0.131568
t = 2.4	0.201022	0.201021	0.191867	0.191867	0.166914	0.166912
t = 2.6	0.189887	0.189885	0.187785	0.187779	0.174984	0.174985
t = 2.8	0.168036	0.168042	0.169772	0.169773	0.164906	0.164909
t = 3.0	0.140544	0.140547	0.143955	0.14396	0.143703	0.143704
t = 3.2	0.110734	0.11073	0.114462	0.114464	0.116436	0.116437
t = 3.4	0.080731	0.080736	0.083975	0.083978	0.086576	0.086585
t = 3.6	0.051844	0.051838	0.054165	0.054153	0.056388	0.056375
t = 3.8	0.02481	0.024819	0.026002	0.025994	0.027265	0.027273
t = 4.0	6.51 $\times \;10^{-16}$	−1.35 $\times \;10^{-5}$	−1.59 $\times \;10^{-15}$	2.75 $\times \;10^{-5}$	−1.23 $\times \;10^{-15}$	2.64 $\times \;10^{-5}$

**Table 15:** The residual error in F when ϕ varies are shown in the table.

Residual Error in F			
**t**	**Residual Error When ϕ = 0.1**	**Residual Error When ϕ = 0.2**	**Residual Error When ϕ = 0.3**
0	−6.89 $\times \;10^{-8}$	−5.75 $\times \;10^{-6}$	1.04 $\times \;10^{-6}$
0.2	2.26 $\times \;10^{-7}$	−5.66 $\times \;10^{-6}$	5.87 $\times \;10^{-6}$
0.4	−1.44 $\times \;10^{-6}$	9.77 $\times \;10^{-6}$	−3.97 $\times \;10^{-7}$
0.6	9.52 $\times \;10^{-7}$	2.77 $\times \;10^{-6}$	−9.14 $\times \;10^{-6}$
0.8	7.88 $\times \;10^{-6}$	2.80 $\times \;10^{-6}$	−2.94 $\times \;10^{-6}$
1	−2.36 $\times \;10^{-6}$	−7.82 $\times \;10^{-6}$	−4.30 $\times \;10^{-6}$
1.2	−8.33 $\times \;10^{-7}$	−3.78 $\times \;10^{-6}$	5.70 $\times \;10^{-7}$
1.4	8.21 $\times \;10^{-7}$	2.15 $\times \;10^{-6}$	−3.74 $\times \;10^{-7}$
1.6	3.36 $\times \;10^{-6}$	1.96 $\times \;10^{-6}$	−4.57 $\times \;10^{-6}$
1.8	5.80 $\times \;10^{-6}$	1.32 $\times \;10^{-6}$	5.16 $\times \;10^{-7}$
2	4.61 $\times \;10^{-6}$	−1.86 $\times \;10^{-6}$	−4.65 $\times \;10^{-6}$
2.2	−4.33 $\times \;10^{-6}$	2.70 $\times \;10^{-6}$	2.36 $\times \;10^{-6}$
2.4	−3.78 $\times \;10^{-6}$	−8.82 $\times \;10^{-6}$	−5.48 $\times \;10^{-6}$
2.6	−6.32 $\times \;10^{-6}$	−7.79 $\times \;10^{-6}$	3.19 $\times \;10^{-6}$
2.8	−5.86 $\times \;10^{-7}$	−6.04 $\times \;10^{-6}$	2.47 $\times \;10^{-6}$
3	−7.84 $\times \;10^{-9}$	5.48 $\times \;10^{-6}$	−7.87 $\times \;10^{-6}$
3.2	−4.60 $\times \;10^{-6}$	3.94 $\times \;10^{-6}$	3.69 $\times \;10^{-6}$
3.4	−3.76 $\times \;10^{-6}$	3.12 $\times \;10^{-6}$	−1.11 $\times \;10^{-6}$
3.6	3.55 $\times \;10^{-7}$	−2.60 $\times \;10^{-6}$	−3.37 $\times \;10^{-6}$
3.8	1.17 $\times \;10^{-6}$	−4.80 $\times \;10^{-6}$	−1.41 $\times \;10^{-6}$
4	−8.76 $\times \;10^{-7}$	−6.28 $\times \;10^{-6}$	−4.47 $\times \;10^{-6}$

**Table 16:** The residual error between RK4 and LMBNN in the outputs of H.

Residual Error in H			
**t**	**Residual Error When ϕ = 0.1**	**Residual Error When ϕ = 0.2**	**Residual Error When ϕ = 0.3**
0	−1.38 $\times \;10^{-6}$	−3.57 $\times \;10^{-6}$	1.06 $\times \;10^{-6}$
0.2	−5.09 $\times \;10^{-7}$	−4.04 $\times \;10^{-6}$	3.49 $\times \;10^{-6}$
0.4	−2.59 $\times \;10^{-6}$	6.17 $\times \;10^{-6}$	4.49 $\times \;10^{-7}$
0.6	2.84 $\times \;10^{-7}$	7.50 $\times \;10^{-7}$	−2.63 $\times \;10^{-6}$
0.8	5.39 $\times \;10^{-6}$	1.44 $\times \;10^{-6}$	5.63 $\times \;10^{-7}$
1	−8.18 $\times \;10^{-7}$	−2.79 $\times \;10^{-6}$	6.30 $\times \;10^{-7}$
1.2	1.46 $\times \;10^{-8}$	−3.01 $\times \;10^{-7}$	7.21 $\times \;10^{-7}$
1.4	−2.48 $\times \;10^{-8}$	−1.67 $\times \;10^{-6}$	−8.33 $\times \;10^{-7}$
1.6	5.24 $\times \;10^{-7}$	−3.24 $\times \;10^{-6}$	2.79 $\times \;10^{-6}$
1.8	−2.16 $\times \;10^{-6}$	−1.28 $\times \;10^{-6}$	1.95 $\times \;10^{-7}$
2	−1.68 $\times \;10^{-6}$	1.22 $\times \;10^{-6}$	3.40 $\times \;10^{-6}$
2.2	−1.12 $\times \;10^{-7}$	−3.34 $\times \;10^{-6}$	−6.20 $\times \;10^{-6}$
2.4	3.98 $\times \;10^{-6}$	6.37 $\times \;10^{-6}$	5.24 $\times \;10^{-6}$
2.6	2.05 $\times \;10^{-7}$	7.08 $\times \;10^{-6}$	−9.05 $\times \;10^{-6}$
2.8	−5.21 $\times \;10^{-6}$	1.68 $\times \;10^{-6}$	−1.07 $\times \;10^{-6}$
3	−5.63 $\times \;10^{-6}$	−8.12 $\times \;10^{-6}$	2.08 $\times \;10^{-6}$
3.2	2.52 $\times \;10^{-6}$	−4.18 $\times \;10^{-6}$	−9.71 $\times \;10^{-7}$
3.4	−3.59 $\times \;10^{-6}$	−2.92 $\times \;10^{-6}$	−1.03 $\times \;10^{-5}$
3.6	5.45 $\times \;10^{-6}$	1.09 $\times \;10^{-5}$	1.18 $\times \;10^{-5}$
3.8	−6.66 $\times \;10^{-6}$	5.54 $\times \;10^{-6}$	−7.04 $\times \;10^{-6}$
4	5.78 $\times \;10^{-6}$	−1.09 $\times \;10^{-5}$	−1.10 $\times \;10^{-5}$

**Table 17:** The residual error in Θ when volume fraction ϕ varies.

Residual Error in Θ			
**t**	**Residual Error When ϕ = 0.1**	**Residual Error When ϕ = 0.2**	**Residual Error When ϕ = 0.3**
0	−9.21 $\times \;10^{-6}$	−1.17 $\times \;10^{-5}$	−1.52 $\times \;10^{-5}$
0.2	−2.88 $\times \;10^{-7}$	−2.39 $\times \;10^{-6}$	8.44 $\times \;10^{-6}$
0.4	8.56 $\times \;10^{-7}$	8.62 $\times \;10^{-6}$	3.85 $\times \;10^{-6}$
0.6	2.76 $\times \;10^{-6}$	6.57 $\times \;10^{-6}$	−1.01 $\times \;10^{-6}$
0.8	−2.58 $\times \;10^{-6}$	−3.67 $\times \;10^{-6}$	−2.56 $\times \;10^{-6}$
1	2.08 $\times \;10^{-6}$	−2.62 $\times \;10^{-6}$	6.56 $\times \;10^{-6}$
1.2	4.22 $\times \;10^{-6}$	−3.68 $\times \;10^{-6}$	−3.66 $\times \;10^{-6}$
1.4	1.99 $\times \;10^{-6}$	−1.32 $\times \;10^{-6}$	−3.82 $\times \;10^{-7}$
1.6	4.59 $\times \;10^{-6}$	−1.18 $\times \;10^{-6}$	8.73 $\times \;10^{-6}$
1.8	−2.58 $\times \;10^{-6}$	−5.09 $\times \;10^{-6}$	−9.82 $\times \;10^{-6}$
2	−6.68 $\times \;10^{-6}$	−2.64 $\times \;10^{-6}$	9.86 $\times \;10^{-6}$
2.2	6.25 $\times \;10^{-6}$	−5.51 $\times \;10^{-6}$	−7.37 $\times \;10^{-6}$
2.4	5.30 $\times \;10^{-6}$	7.41 $\times \;10^{-6}$	4.37 $\times \;10^{-6}$
2.6	−4.81 $\times \;10^{-7}$	4.50 $\times \;10^{-6}$	−1.19 $\times \;10^{-5}$
2.8	−2.07 $\times \;10^{-6}$	3.35 $\times \;10^{-6}$	5.52 $\times \;10^{-7}$
3	−5.27 $\times \;10^{-6}$	−6.20 $\times \;10^{-6}$	3.29 $\times \;10^{-6}$
3.2	5.32 $\times \;10^{-8}$	−3.95 $\times \;10^{-6}$	−5.06 $\times \;10^{-7}$
3.4	−1.81 $\times \;10^{-6}$	−1.84 $\times \;10^{-6}$	−7.95 $\times \;10^{-6}$
3.6	3.75 $\times \;10^{-6}$	7.71 $\times \;10^{-6}$	9.03 $\times \;10^{-6}$
3.8	−4.51 $\times \;10^{-6}$	3.90 $\times \;10^{-6}$	−5.38 $\times \;10^{-6}$
4	5.54 $\times \;10^{-6}$	−1.18 $\times \;10^{-5}$	−1.21 $\times \;10^{-5}$

**Table 18:** The residual error in Φ are shown in the following the table.

Residual Error in Φ			
**t**	**Residual Error When ϕ = 0.1**	**Residual Error When ϕ = 0.2**	**Residual Error When ϕ = 0.3**
0	8.62 $\times \;10^{-6}$	2.56 $\times \;10^{-6}$	1.50 $\times \;10^{-5}$
0.2	−3.20 $\times \;10^{-6}$	1.63 $\times \;10^{-6}$	−3.56 $\times \;10^{-6}$
0.4	1.09 $\times \;10^{-7}$	5.54 $\times \;10^{-6}$	−1.17 $\times \;10^{-5}$
0.6	5.06 $\times \;10^{-6}$	−6.33 $\times \;10^{-6}$	2.49 $\times \;10^{-6}$
0.8	5.29 $\times \;10^{-6}$	2.26 $\times \;10^{-6}$	1.00 $\times \;10^{-5}$
1	3.24 $\times \;10^{-6}$	−3.88 $\times \;10^{-6}$	−3.43 $\times \;10^{-6}$
1.2	−3.15 $\times \;10^{-6}$	−1.04 $\times \;10^{-6}$	2.45 $\times \;10^{-6}$
1.4	1.54 $\times \;10^{-6}$	−5.99 $\times \;10^{-6}$	−5.66 $\times \;10^{-6}$
1.6	−4.82 $\times \;10^{-6}$	−3.24 $\times \;10^{-6}$	−4.33 $\times \;10^{-6}$
1.8	−7.96 $\times \;10^{-7}$	2.23 $\times \;10^{-6}$	1.03 $\times \;10^{-5}$
2	4.41 $\times \;10^{-6}$	4.83 $\times \;10^{-6}$	−5.61 $\times \;10^{-6}$
2.2	−6.79 $\times \;10^{-6}$	2.33 $\times \;10^{-7}$	−8.62 $\times \;10^{-7}$
2.4	1.39 $\times \;10^{-6}$	4.53 $\times \;10^{-7}$	2.45 $\times \;10^{-6}$
2.6	1.56 $\times \;10^{-6}$	5.67 $\times \;10^{-6}$	−5.85 $\times \;10^{-7}$
2.8	−5.62 $\times \;10^{-6}$	−7.14 $\times \;10^{-7}$	−2.98 $\times \;10^{-6}$
3	−2.52 $\times \;10^{-6}$	−4.74 $\times \;10^{-6}$	−1.38 $\times \;10^{-6}$
3.2	4.40 $\times \;10^{-6}$	−1.72 $\times \;10^{-6}$	−1.43 $\times \;10^{-6}$
3.4	−4.35 $\times \;10^{-6}$	−3.41 $\times \;10^{-6}$	−9.23 $\times \;10^{-6}$
3.6	6.31 $\times \;10^{-6}$	1.20 $\times \;10^{-5}$	1.28 $\times \;10^{-5}$
3.8	−8.53 $\times \;10^{-6}$	7.89 $\times \;10^{-6}$	−8.32 $\times \;10^{-6}$
4	1.35 $\times \;10^{-5}$	−2.75 $\times \;10^{-5}$	−2.64 $\times \;10^{-5}$

## Data Availability

The data that support the findings of this study are available from the corresponding author upon reasonable request.

## Abbreviations

The following abbreviations are used in this manuscript:

ANN	Artificial neural network
RK4	Runge-kutta order four technique
LMBNN	Levenberg–Marquardt backpropagation neural network
mse	Mean squared error
